# Initial or continuous coculture with umbilical cord-derived mesenchymal stromal cells facilitates in vitro expansion of human regulatory T-cell subpopulations

**DOI:** 10.1093/stcltm/szaf012

**Published:** 2025-06-14

**Authors:** Qifeng Ou, Sarah Cormican, Rachael Power, Sarah Hontz, Shirley A Hanley, Md Nahidul Islam, Georgina Shaw, Laura M Deedigan, Emma Horan, Stephen J Elliman, Barbara Fazekas, Janusz Krawczyk, Neema Negi, Matthew D Griffin

**Affiliations:** Regenerative Medicine Institute (REMEDI) at CÚRAM Research Ireland Centre for Medical Devices, School of Medicine, University of Galway, Galway H19 TK33, Ireland; Regenerative Medicine Institute (REMEDI) at CÚRAM Research Ireland Centre for Medical Devices, School of Medicine, University of Galway, Galway H19 TK33, Ireland; Regenerative Medicine Institute (REMEDI) at CÚRAM Research Ireland Centre for Medical Devices, School of Medicine, University of Galway, Galway H19 TK33, Ireland; Regenerative Medicine Institute (REMEDI) at CÚRAM Research Ireland Centre for Medical Devices, School of Medicine, University of Galway, Galway H19 TK33, Ireland; Flow Cytometry Core Facility, Biomedical Sciences, University of Galway, Galway H19 TK33, Ireland; School of Biological and Chemical Sciences, College of Science and Engineering, University of Galway, Galway H19 TK33, Ireland; Department of Applied Science, Technological University of the Shannon, Limerick V94 EC5T, Ireland; Regenerative Medicine Institute (REMEDI) at CÚRAM Research Ireland Centre for Medical Devices, School of Medicine, University of Galway, Galway H19 TK33, Ireland; Orbsen Therapeutics Ltd., Dangan, Galway H91 A3EF, Ireland; Orbsen Therapeutics Ltd., Dangan, Galway H91 A3EF, Ireland; Orbsen Therapeutics Ltd., Dangan, Galway H91 A3EF, Ireland; Regenerative Medicine Institute (REMEDI) at CÚRAM Research Ireland Centre for Medical Devices, School of Medicine, University of Galway, Galway H19 TK33, Ireland; Biology and Biopharmaceutical Science, Department of Science, South East Technological University, Waterford X91 CF21, Ireland; Regenerative Medicine Institute (REMEDI) at CÚRAM Research Ireland Centre for Medical Devices, School of Medicine, University of Galway, Galway H19 TK33, Ireland; Haematology Department, University Hospital Galway, Saolta University Healthcare Group, Galway H91 YR71, Ireland; Regenerative Medicine Institute (REMEDI) at CÚRAM Research Ireland Centre for Medical Devices, School of Medicine, University of Galway, Galway H19 TK33, Ireland; Department of Chemical Toxicology, Division of Climate and Environment Health, Norwegian Institute of Public Health (Folkehelseinstituttet), 0456 Oslo, Norway; Regenerative Medicine Institute (REMEDI) at CÚRAM Research Ireland Centre for Medical Devices, School of Medicine, University of Galway, Galway H19 TK33, Ireland

**Keywords:** cell therapy, cell manufacturing, culture expansion, clinical translation, immunological diseases, mesenchymal stromal cells, regulatory T cells, subpopulations, yield

## Abstract

Clinical trials have demonstrated the safety and potential efficacy of ex vivo expanded regulatory T cells (Tregs) for immune-mediated diseases. Nonetheless, achieving consistent and timely Treg yield and purity remains challenging. We aimed to evaluate the potential to enhance culture expansion of primary human total Treg (CD4^+^/CD25^+^/CD127^lo^) and Treg subpopulations through coculture with human umbilical cord-derived mesenchymal stromal cells (hUC-MSCs). In 14- to 21-day anti-CD3/anti-CD28-, interleukin-2-, and rapamycin-containing cultures, fluorescence-activated cell sorting (FACS)-purified total Treg underwent 4-fold greater expansion following hUC-MSC coculture. Potency to suppress T effector cell (Teff) proliferation was equivalent for hUC-MSC-cocultured and control Tregs and correlated with the expression of HLA-DR, CD39, and inducible costimulator (ICOS). The impact of hUC-MSC coculture on ex vivo expansion of 3 FACS-purified Treg subpopulations [CD45RA^+^ (Subtype I), CD45RA^−^HLA-DR^+^ (Subtype II), and CD45RA^−^HLA-DR^−^ (Subtype III)] was then investigated. Both initial and continuous hUC-MSC coculture yielded significantly higher fold expansion of each Treg subpopulation compared to control. However, the magnitude of enhancement was substantially greater for non-naive (Subtypes II and III) than for naive (Subtype I) Treg. Coculture with hUC-MSC increased HLA-DR expression of all 3 expanded Treg subpopulations while maintaining comparable Teff suppressive potency. For non-naive Treg (Subtypes II and III), both initial and continuous hUC-MSC coculture also increased the final %Foxp3^+^ and %Helios^+^. Thus, coculture with clinical-grade hUC-MSC substantially enhances the ex vivo yield, preserves the suppressive potency, and modulates HLA-DR expression of FACS-purified Treg subpopulations with greatest effect on non-naive (CD45RA^−^) Treg. The findings have potential to facilitate identification, functional characterization, and manufacturing of Treg subpopulations with distinct therapeutic benefits.

## Graphical Abstract

**Figure:**
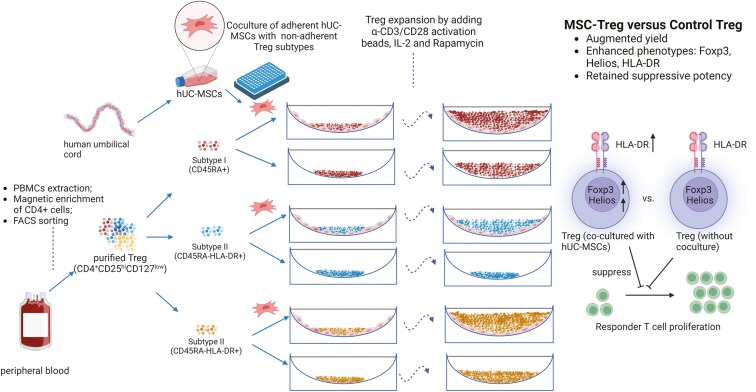
Coculture with human umbilical cord-derived mesenchymal stromal cells (hUC-MSCs) enhances the ex vivo expansion of 3 subtypes (CD45RA^+^, CD45RA^−^HLA-DR^−^, and CD45RA^−^HLA-DR^+^) of regulatory T cells (Tregs) from human peripheral blood. This enhancement includes higher yield, increased expression of Foxp3, Helios, and HLA-DR, and similar suppressive potency compared to Treg cultured alone. MSC-Treg = hUC-MSC-cocultured Treg.

**Significance statement**

Regulatory T cells (Tregs) control overactivity of the immune system. They can be extracted from the blood of individuals with diseases involving overactive immunity, stimulated to divide and increase in number, and then returned to the patient. One challenge to this therapy is that the number of Tregs produced may be too small for an effective dose. This challenge is even greater if we wish to use Treg subtypes that are only present in very small numbers in the blood. Our study helps to overcome that challenge by showing that coculturing small numbers of blood-purified Tregs with mesenchymal stromal cells (MSCs), a type of stem cell, increases Treg numbers without affecting their function.

**Lessons learned**

• Human regulatory T cells (Tregs), which are a promising cell therapy for diseases involving overactive immune responses, could be produced in higher numbers from blood samples when combined with mesenchymal stromal cells (MSCs), a type of human stem cell.
• When human Tregs were subdivided into different types that may have different treatment effects, combining them with human MSCs during the expansion stage had a greater effect on the cell yield of "experienced" compared to "naive" Tregs.
• The Tregs that were produced in culture from blood samples combined with human MSCs had the same ability to dampen the activity of immune cells as those produced in the usual way.
• Because MSCs are also used to treat patients with various diseases, this approach could be adopted to increase the production of Treg subtypes that are only present in low numbers in the blood for treatment of immunological diseases.

## Introduction

Regulatory T cells (Tregs) are a subset of CD4^+^ T cells that are characterized by high surface expression of the IL-2 receptor alpha chain CD25,^1^ low surface expression of the IL-7 receptor CD127,^2^ and intracellular expression of the transcription factor forkhead box P3 (FoxP3).^3^ Functionally, Tregs serve to negatively regulate adaptive and innate immune responses to either self- or non-self-antigens by a variety of mechanisms.^4^ This underlies their essential role to prevent autoimmunity^5^ and excessive inflammation as well as their capacity to partly mitigate rejection of allogeneic organ and tissue transplants.^6,7^ Following their initial discovery and the development of culture conditions that allow for their effective expansion ex vivo, Tregs have been viewed as a potentially transformative adoptive cell therapy for the prevention or treatment of diseases involving adverse immune responses.^6,8^ In recent years, the safety and feasibility of autologous polyclonal or antigen-specific culture-expanded Tregs have been borne out in multiple early-phase clinical trials involving patients with autoimmune diseases and recipients of organ transplants.^9,10^ Although a number of these trials have provided preliminary signals of clinical benefit, most have also revealed significant obstacles to the achievement of consistent, unequivocal efficacy.^11^

Protocols for the manufacture of good manufacturing practice (GMP)-compliant Treg products from individual patients have been successfully developed by clinical centers across the globe.^12-14^ However, achieving consistent and timely yield and purity of ex vivo expanded Tregs remains an important challenge.^11,15,16^ The most commonly used source for Treg-based cell products is peripheral blood leukocytes/mononuclear cells of which Tregs typically constitute <1%-3% of total cells—necessitating cell collection from a relatively large volume of blood and/or prolonged culture expansion periods. Indeed, in some clinical trial reports, failure to generate adequate Treg number from individual samples has limited the number of treated participants.^17,18^ A second important challenge lies in the purity of Tregs both within the primary source cells and in the final culture-expanded cell product. For example, primary Treg isolation is often performed by magnetic enrichment-based positive selection of CD4- and CD25-expressing cells with or without negative selection of CD127-high cells—an approach that provides higher cell yield and is readily adapted to GMP conditions but may result in contamination with nonregulatory T effector cells (Teffs)^19,20^ or other lymphocytes needing repeated depletion during expansion.^10^ In comparison, the use of fluorescence-activated cell sorting (FACS) based on multiple cell surface markers has the potential to substantially increase initial Treg purity but typically results in lower initial numbers of viable cells.^21,22^

An additional important limiting factor to the clinical efficacy of current Treg therapies is the extent of functional and phenotypic heterogeneity that is present within CD4^+^/CD25^+^/CD127^lo^/FoxP3^+^ Tregs. It is now well recognized that this population includes both naive and non-naive (antigen-experienced) subsets as well as functionally distinct subpopulations characterized by differential expression of a range of proteins including CD39,^23^ Helios,^24^ inducible costimulator (ICOS),^25^ cytotoxic T lymphocyte antigen (CTLA)-4 (CD152),^26^ Class II MHC,^27^ and others.^28^ It is highly likely that the clinical benefit of Treg therapy for specific immune-mediated diseases or transplant complications can be improved by enriching the cell product for Treg subpopulations that mediate the most relevant suppressive mechanisms. However, this strategy has not been robustly pursued to date as a result of technical limitations to the effective propagation of purified Treg subpopulations and limited knowledge of their distinctive functional properties following ex vivo expansion.^29,30^

One approach to overcoming such obstacles to the manufacture of effective Treg therapies is to harness the supportive interactions that are known to occur between Tregs and stromal cells. Indeed, mesenchymal stromal cell (MSC)/Treg interplay has frequently been implicated in the immune modulatory properties of MSC-based cell therapies.^7,31,32^ In this regard, Reading et al. recently reported that coculture of FACS-purified human Tregs with multipotent adult progenitor cells (MAPCs)—a bone marrow-derived stromal cell product—in the context of a GMP-compatible ex vivo Treg expansion protocol, resulted in substantial increase in the yields of potently suppressive Tregs as well as distinctive transcriptional alterations.^33^ In the current study, we first demonstrate that the yield from culture expansion of primary CD4^+^/CD25^+^/CD127^lo^ human Tregs is significantly enhanced following an initial coculture with CD362-selected human umbilical cord-derived MSCs (hUC-MSCs). The rationale for using CD362-selected MSCs in this study is derived from their potential for prospective isolation from multiple source tissues based on expression of CD362 (syndecan-2), their previously reported therapeutic benefits in multiple preclinical models of inflammatory/immune-mediated diseases, and their documented safety as a GMP-grade cell therapy product in early-phase clinical trials.^34-38^ Subsequently, we investigated the differential effects of hUC-MSC coculture on ex vivo expansion of 3 different FACS-purified Treg subpopulations. By this approach, we aimed to gain insights into the potential for this method to facilitate the manufacture of refined Treg immune modulatory therapies in the future.

## Materials and methods

### Isolation of human Tregs and Treg subpopulations

Tregs and Teffs were isolated from peripheral blood samples collected from healthy adult volunteers through the HRB Clinical Research Facility Galway, following informed consent and according to a protocol approved by the Galway University Hospitals Clinical Research Ethics Committee (protocol number C.A.1921). A total of 50 mL of peripheral venous blood was collected from each donor in 10 mL ethylenediaminetetraacetic acid (EDTA) Vacutainer tubes (BD Medical Supplies, Crawley, UK) by standard venipuncture technique, and samples were processed within 2 hours of collection. Peripheral blood mononuclear cells (PBMCs) were isolated using Ficoll-Plaque Plus density gradient centrifugation (GE Healthcare, Little Chalfont, UK) as previously described.^39^ Single-cell suspensions were then obtained by filtering through a 30-µm pre-separation filter (Miltenyi Biotec, Bergisch Gladbach, Germany). The cells were pelleted by centrifugation and resuspended in MACS buffer [DPBS (Gibco), 0.5% bovine serum albumin, 2 mM EDTA] followed by enrichment of CD4^+^ T cells by magnetic cell sorting using anti-human CD4 microbeads, MS columns, and an OctoMACS separator according to the manufacturer’s instructions (Miltenyi Biotec). Thereafter, enriched CD4^+^ T cells (10-20 × 10^6^) were suspended in FACS buffer and labeled with mouse anti-human CD4 FITC, CD25 PE-Cy7, and CD127 PE monoclonal antibodies. The stained cells were then washed and resuspended in SORT buffer [DPBS, 1% fetal bovine serum (FBS; Sigma-Aldrich, USA), 25 mM HEPES solution, and 2 mM EDTA (Sigma-Aldrich)]. The viability dye Draq7 (BioLegend, UK Ltd) was added immediately prior to cell sorting, and viable Tregs (Draq7^−^/CD4^+^/CD25^hi^/CD127^lo^) were then sorted using a BD FACS Aria II high-speed cell sorter and BD FACS Diva v6 software (BD Biosciences) according to the gating strategy shown in Supplementary Figure S1. For Treg subpopulation sorting, 10-20 × 10^6^ MACS-enriched CD4^+^ T cells suspended in FACS buffer were labeled with mouse anti-human CD4 FITC, CD25 PE-Cy7, CD127 PE, CD 45RA APC, and HLA-DR eFluor-450 followed by washing, resuspension in SORT buffer, and sorting into 3 subtypes according to the gating strategy shown in Figure 5A. Controls used included single stain compensation controls and fluorescence minus one (FMO) gating controls. Details of antibody products used for FACS and flow cytometry-based analyses for the study are provided in Supplementary Table S3.

### Culture and characterization of hUC-MSCs

Anti-CD362-selected hUC-MSCs were cultured from ethically sourced human umbilical cord tissue obtained from Tissue Solutions Ltd. (Glasgow, UK). Primary isolation and expansion cultures of CD362-selected hUC-MSCs were carried out as previously described.^37,40^ Cryopreserved vials of passage (P)1-P2 hUC-MSCs were thawed and cultured to 80%-90% confluence in minimum essential medium (MEM)-α, supplemented with 10% FBS and 1% penicillin-streptomycin in tissue culture flasks in a humidified incubator at 37 °C, 20% O_2_, and 5% CO_2_. The P2-P3 hUC-MSCs were then lifted by trypsinization, divided into aliquots, and cryopreserved until use in Treg cocultures. In preparation for initiation of cocultures, P3-P6 hUC-MSCs were thawed and placed in culture for 1-2 days before use.

Surface expression by CD362-selected hUC-MSCs of relevant positive and negative MSC markers^41^ was analyzed by flow cytometry. In brief, 0.1 mL of cell suspension (1 × 10^5^ cells/sample) was incubated with optimized concentrations of fluorochrome-coupled monoclonal antibodies for 30 minutes in the dark at 4 °C. The following antibodies (all from R&D Systems, Minneapolis, MN, USA) were used: CD90-PE, HLA-DR-PerCP, CD3-APC, CD73-APC, CD105-AF488, CD31-FITC, and CD326-PE. To set the background fluorescence levels, appropriate isotype-matched controls were used in conjunction with FMO controls. Post-incubation, cells were washed twice, resuspended in 0.2 mL FACS buffer, and analyzed using a MACSQuant Analyzer 10 (Miltenyi Biotech) and FlowJo software V10.10 (Tree Star, Inc.). As shown by representative histograms in Supplementary Figure S10A, the hUC-MSCs used for coculture experiments were highly positive for CD73, CD90, and CD105 and lacked expression of hematopoietic markers CD3 and HLA-DR as well as endothelial and epithelial markers CD31 and CD326.

Trilineage differentiation of P6 CD362-selected hUC-MSCs was assessed using well-established in vitro differentiation protocols followed by staining for adipogenic (Oil Red O), osteogenic (Alizarin Red), and chondrogenic (Alcian Blue) differentiation as previously described.^42^ As shown in representative photomicrographs in Supplementary Figure S10B, hUC-MSCs subjected to the relevant differentiation conditions for 2 weeks demonstrated staining consistent with adipogenic and osteogenic potential compared to control cultures. However, hUC-MSC pellet cultures under chondrogenic conditions were larger than control cultures but did not stain more intensely with Alcian Blue. These mesenchymal lineage differentiation characteristics were consistent with previous reports for human MSCs derived from umbilical cord.^43^

### Expansion of Tregs and establishment of coculture system

**Figure 1.**
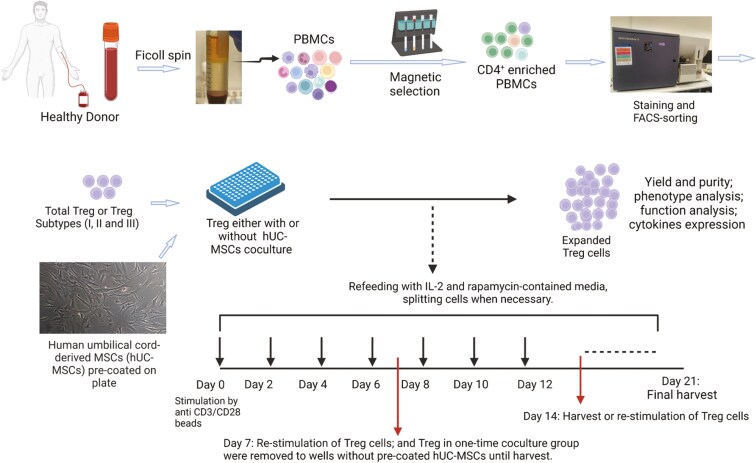
Protocol for ex vivo expansion of purified human regulatory T cells (Tregs) in the presence or absence of human umbilical cord-derived mesenchymal stromal cells (hUC-MSCs): Illustration summarizing the steps followed in purifying human Tregs and Treg subpopulations for the study (upper panel) and the details of an ex vivo culture expansion protocol of the purified Tregs in the presence or absence of hUC-MSCs.

Freshly sorted Tregs and Treg subpopulations were seeded into 96-well U-bottom tissue culture plates (Sarstedt, Germany) at 12.5 or 25 × 10^3^ cells/well and were activated using MACSiBeads (Treg expansion kit, Miltenyi) or Human T-Activator CD3/CD28 Dynabeads (ThermoFisher) at bead:cell ratios of 4:1 (see also Figure 1 and Supplementary Table S2). Cultures were established in complete Treg expansion medium consisting of TexMACS Medium (Miltenyi) supplemented with 5% human AB serum (Sigma-Aldrich, USA), 1% penicillin-streptomycin (Sigma-Aldrich, Israel), 500 IU/mL human recombinant IL-2 (Miltenyi), and 200 nmol/mL rapamycin (Miltenyi). For hUC-MSC coculture conditions (MSC-Treg), individual tissue culture plate wells were preseeded with 5 × 10^3^ live hUC-MSCs suspended in 50 μL Treg expansion medium and placed in a tissue culture incubator for 6-8 hours prior to addition of Tregs/activation beads to final volumes of 200 μL/well and resulting in hUC-MSCs:Treg ratios of 1:5 or 1:2.5 (Supplementary Table S2). For control culture conditions (Ctrl-Treg), Treg/activation beads only were added to final volumes of 200 μL/well. All cultures were carried out in a humidified incubator at 37 °C, 20% O_2_, and 5% CO_2_.

During the expansion period, medium was exchanged every 2 days (or sooner if medium color changed) by gently removing 150 μL from each well and replacing with 150 μL of complete Treg medium. When cell number exceeded 2 × 10^5^/well, the contents of all wells for a given condition were pooled and transferred to the appropriate number of wells of a new 96-well U-bottom plate to maintain the concentration of 5 × 10^4^ cells/well in complete Treg medium. At culture day 7, Tregs were removed from coculture wells and were reactivated by depleting old activator beads using an MACSiMAG Separator (Miltenyi), following which the cells were mixed with fresh beads at bead:cell ratio of 1:1. For culture conditions in which Tregs were activated in the presence of hUC-MSCs for the first expansion cycle only [MSC-Treg (Once)], no further hUC-MSCs were added to the wells into which Tregs were transferred for reactivation. For conditions in which Tregs were cocultured with hUC-MSCs throughout the entire expansion period [MSC-Treg (Constant)], the appropriate number of wells of the reactivation plates was preseeded with hUC-MSCs for 6 hours. For the initial experiments involving culture expansion of total Tregs, the cells were reactivated in the same manner for another 7-day cycle and were then collected at day 21 for calculation of fold increase and for phenotypic and functional analyses. For experiments involving culture expansion of Treg subpopulations, the cells were cultured in the same condition as total Tregs except that cells were collected for analyses at day 14.

### Analysis of yields and phenotypes of culture-expanded Tregs

After depletion of activator beads by MACSiMAG Separator (Miltenyi, 130-092-168), either total Tregs or Treg subpopulations were counted by Trypan blue exclusion using a hemocytometer. Fold expansion from the originally seeded numbers of cells was then calculated for each condition. Once counted, the expanded Tregs were either cryopreserved or were used immediately for phenotypic characterization by flow cytometry. For cryopreservation, up to 3 × 10^6^ Tregs were resuspended in 500 μL of Treg expansion medium in 1.5 mL CryoTube vials (Thermo Fisher) following which an equal volume of medium containing 20% DMSO was added drop-by-drop. The cell suspensions were then mixed, and the sealed vials were stored at −80 °C in a freezing container for 2 days before transfer to liquid N_2_. For analysis of expression profiles for relevant surface and intracellular proteins, a 12-color flow cytometric panel was developed, which included a fixable viability dye (Zombie Red, BioLegend), 9 surface markers [CD4 FITC, CD25 PE-Cy7, CD127 BV785 (BioLegend), HLA-DR eFluor-450, CD45RA APC, CD45RO PerCP, CD39 PerCP-eFluor-710, ICOS BV510, and CCR6 PE], and 3 intracellular markers (Helios PE-eFluor-610, Foxp3 R718, and CTLA-4 BUV805). Fixation and permeabilization were accomplished using the eBioscience Intracellular Fixation & Permeabilization Buffer Set (Thermo Fisher Scientific) according to the manufacturer’s instructions. After step-by-step staining, the samples were analyzed on a spectral flow cytometer (Cytek Biosciences Northern Lights 3000). Unmixing of the 12-color panel was based on single staining controls using Compensation Beads (OneComp eBeads, Thermo Fisher Scientific). FMO controls were used to set the position of the gates. Subsequently, data files were analyzed using FlowJo 7.6.5 software (TreeStar Inc., Olten, Switzerland).

Four healthy donor PBMC samples were used for sorting and culture expansion of Treg subpopulations. For 3 of 4 donors, the same multicolor flow cytometry panel and protocol were applied for the purpose of analyzing the relative proportions and surface/intracellular expression profiles of the Treg subpopulations prior to expansion.

### Detection of hUC-MSCs within culture-expanded Treg preparations

To determine whether there was any contamination of ex vivo expanded Tregs with hUC-MSCs at the end of the culture expansion periods, cells were removed from the tissue culture wells by pipetting, washed, and then resuspended in FACS buffer. Anti-CD3/anti-CD28 expansion beads were removed by magnetic precipitation, and the resulting cell suspensions were stained with anti-CD73 FITC and anti-CD90 PE (both from BioLegend) and then analyzed for the proportion of CD73^+^/CD90^+^ cells using a Cytek Northern Light 3000 spectral cytometer and FlowJo 7.6.5 software.

### Suppression assay

**Figure 2.**
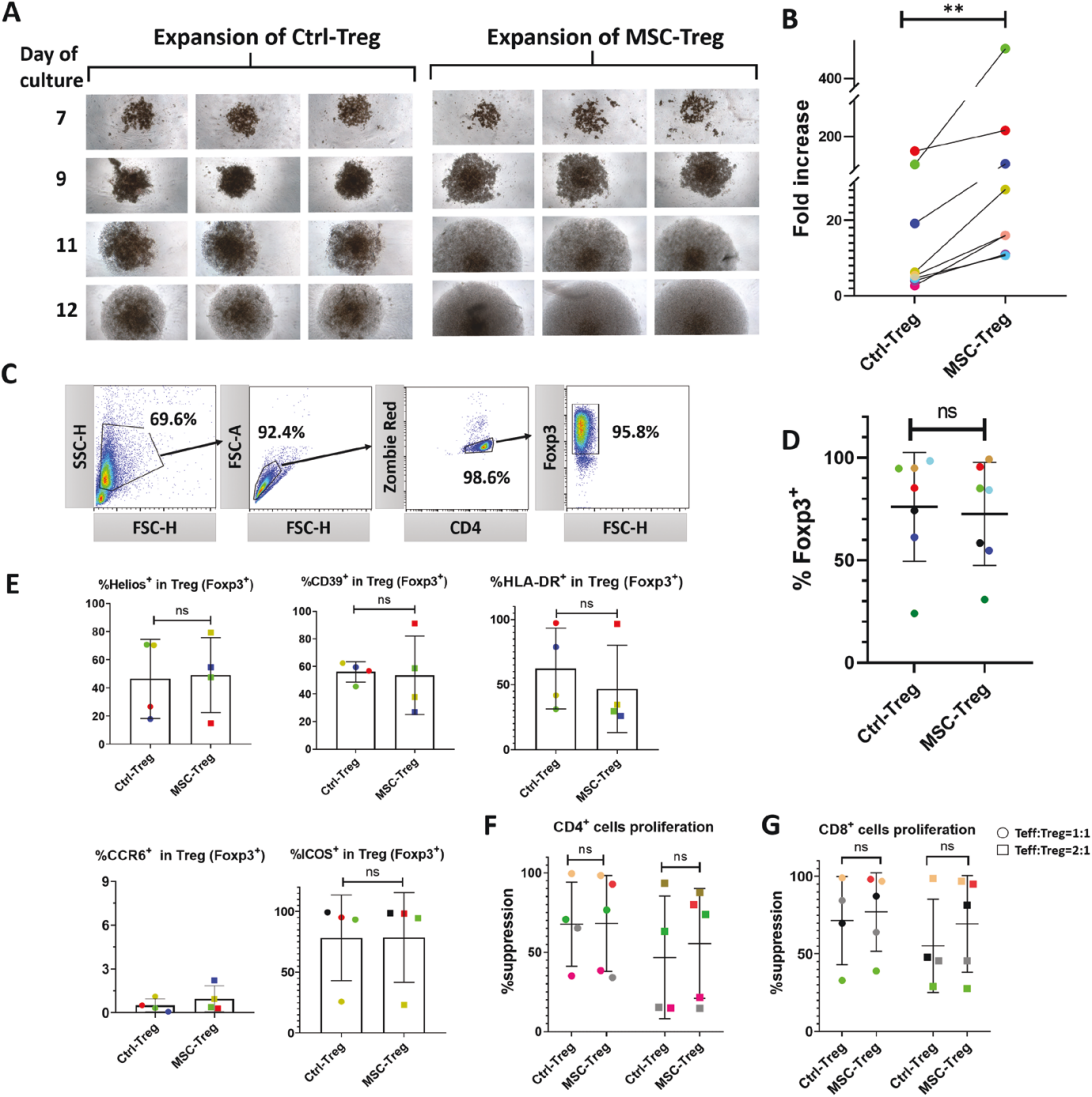
Initial coculture with human umbilical cord-derived mesenchymal stromal cells (hUC-MSCs) increases the yield and preserves the expression of phenotypic markers and suppressive potency of culture-expanded human regulatory T cells (Tregs). Results are shown for human Tregs from up to 7 healthy adult donors following MACSiBeads activation culture expansion in the absence (Ctrl-Treg) and presence (MSC-Treg) of hUC-MSCs during the first expansion cycle. (A) Representative images (inverted microscope, 50×) of individual wells of 96-well Treg expansion cultures at 7, 9, 11, and 12 days for Ctrl-Treg and MSC-Treg. (B) Graph depicting fold increase in cell number from beginning to completion of Ctrl-Treg and MSC-Treg expansion cultures from 7 individual donors. (C) Representative example of the flow cytometry gating strategy for quantifying %Foxp3^+^ in Ctrl-Treg and MSC-Treg. (D) Graph depicting %FoxP3^+^ of Ctrl-Treg and MSC-Treg at completion of expansion cultures from 7 individual donors. (E) Graphs of %^+ve^ among the FoxP3^+^ cells in Ctrl-Treg and MSC-Treg from 4 individual donors for Helios, CD39, CCR6, HLA-DR, and ICOS (%^+ve^ derived from Zombie Red^−^/CD4^+^/Foxp3^+^ cells). (F, G) Graphs depicting the results of suppression assays in which CellTrace Far Red-labeled Ctrl-Treg (*n* = 4 individual donors) and MSC-Treg (*n* = 5 individual donors) were cocultured with polyclonally activated CellTrace Violet (CTV)-labeled primary human CD4^+^ (F) and CD8^+^ (G) T effector cells (Teffs) at Teff:Treg ratios of 1:1 and 2:1. Results are expressed as %suppression of Teff proliferation based on CTV dilution and compared to proliferation of Teffs activated in the absence of Tregs. For all graphs, the results for Tregs from individual donors are distinguished by color, while horizontal strokes and error bars represent mean ± SEM. Statistical analyses for all paired comparisons between Ctrl-Treg and MSC-Treg (B, D, E, F, and G) were performed by Wilcoxon signed-rank test.

The suppressive potency of Tregs and Treg subpopulations culture-expanded under various conditions was quantified by coculture of Tregs with CD4^+^ and CD8^+^ Teffs at Teff:Treg ratios of 1:0 (Teff alone—control condition), 1:1, 2:1, 4:1, and 8:1. Teffs were enriched from PBMCs of healthy adult donors by magnetic bead positive selection using anti-human CD4 or CD8 MicroBeads (Miltenyi) according to the manufacturer’s instructions. For the suppression assays, the Tregs and Teffs were derived from different donors. Culture-expanded Tregs were prestained with CellTrace Far Red (CTFR, Thermo Fisher Scientific), while Teffs were prestained with CellTrace Violet (CTV, Thermo Fisher Scientific) using labeling protocols that were optimized for fluorescence intensity and cell viability. Following the labeling protocols, both CTFR-Tregs and CTV-Teffs were washed and resuspended in RPMI-1640 medium supplemented with 10% FBS and 1% penicillin-streptomycin. To initiate suppression assays, 5 × 10^4^ CTV-Teffs were added to individual wells of a 96-well U-bottom tissue culture plate and activated by the addition of Human T-Activator CD3/CD28 Dynabeads at a 1:4 bead:Teff ratio and of CTFR-Tregs or medium alone to achieve the relevant Treg:Teff ratios, with a final medium volume of 200 μL/well. Triplicate wells were set up for each condition. The plates were cultured for 3-4 days in a tissue culture incubator at 37 °C, 20% O_2_, and 5% CO_2_, following which the cells from each well were collected by pipetting and stained with 7-AAD viability staining (BioLegend) for 30 seconds prior to being analyzed by spectral flow cytometry (Cytek, Northern Light 3000). Data files were analyzed using FlowJo 7.6.5 software (TreeStar Inc.) with Tregs and Teffs fully distinguished on the basis of their respective fluorescent labels (as shown in Figure 2F and G). Suppressive potency was calculated using the division index (DI) of CTV-Teff and was expressed as the calculated percentage of suppression [%Suppression (DI) = 100 − [(DI of the 1:X)/(DI of 0:1)] × 100, where 1:X denotes cocultures with Treg and 0:1 signifies cocultures with responder T cells alone].

### Statistical analysis

GraphPad Prism version 6 (La Jolla, CA, USA) was used for all the experimental statistical analysis. Datasets for individual conditions were analyzed for normality using the Shapiro-Wilk normality test. Paired or unpaired Student’s *t*-tests and Wilcoxon signed-rank tests were used for parametric and nonparametric calculations as appropriate. Multiple comparisons were performed by 1-way or 2-way analysis of variance (ANOVA) followed by Sidak’s multiple comparisons test. Correlation analyses were performed by linear regression using Pearson correlation coefficient. Data are presented as appropriate as mean ± SEM or as median (range). Significance was assigned to *P* <.05 and is denoted in the figures as *P < .05, **P < .01, and ***P < .001.

## Results

### Coculture with hUC-MSCs increases the yield of human Tregs in expansion cultures without reduction in purity and function

Based on a 4-color panel including a viability dye (Draq7) and 3 surface markers (CD4, CD25, and CD127), Tregs (Draq7^−^/CD4^+^/CD25^hi^/CD127^lo^) were purified by FACS from 50 mL peripheral blood samples of 7 healthy donors (Figure 1, upper panel, with gating strategy shown in Supplementary Figure S1). As presented in Supplementary Table S1, the average presort proportion of Tregs among CD4^+^ cells for these samples was 3.5 ± 0.5%, and the yields of purified Tregs varied from 1.1-6.0 × 10^5^ cells (average 4.3 ± 1.9 × 10^5^ cells). Following a 2- to 3-week ex vivo expansion protocol (summarized in Figure 1, lower panel, and described in detail in Materials and Methods), using MACSiBeads as the primary stimulus, the appearance of the expanding cell clusters in each well became visibly larger from approximately day 9 onward (Figure 2A) for Tregs that were cocultured with hUC-MSCs for the first expansion cycle (MSC-Treg) in comparison to Treg that were cultured alone (Ctrl-Treg). Consistent with this, as shown in Figure 2B, the magnitude of expansion was higher for MSC-Treg compared to Ctrl-Treg (112.7 ± 180.3 vs. 30.4 ± 46.75-fold increase), resulting in an average 3.9 ± 0.7-fold higher cell yield.

When Treg purity at the end of the expansion culture protocol was evaluated based on intracellular flow cytometry for FoxP3 (Figure 2C), substantial interdonor variability was observed, but the %FoxP3^+^ was closely comparable for MSC-Treg and Ctrl-Treg from each donor (Figure 2D, 72.6 ± 25.15% vs. 76.1 ± 26.5%). The proportions expressing functionally relevant markers including intracellular Helios and surface CD39, Class II MHC (HLA-DR), CCR6, and ICOS were also not different between MSC-Treg and Ctrl-Treg (Figure 2E, evaluated for Tregs from 4/7 donors; representative dot plots are shown in Supplementary Figure S2A). Similarly, in coculture assays with polyclonally stimulated CD4 and CD8 Teffs, MSC-Treg and Ctrl-Treg derived from 5 and 4 individual donors, respectively, demonstrated variable but closely comparable suppressive potency at 1:1 and 2:1 Teff:Treg ratios (Figure 2F and G; representative dot plots are shown in Supplementary Figure S2B).

**Figure 3.**
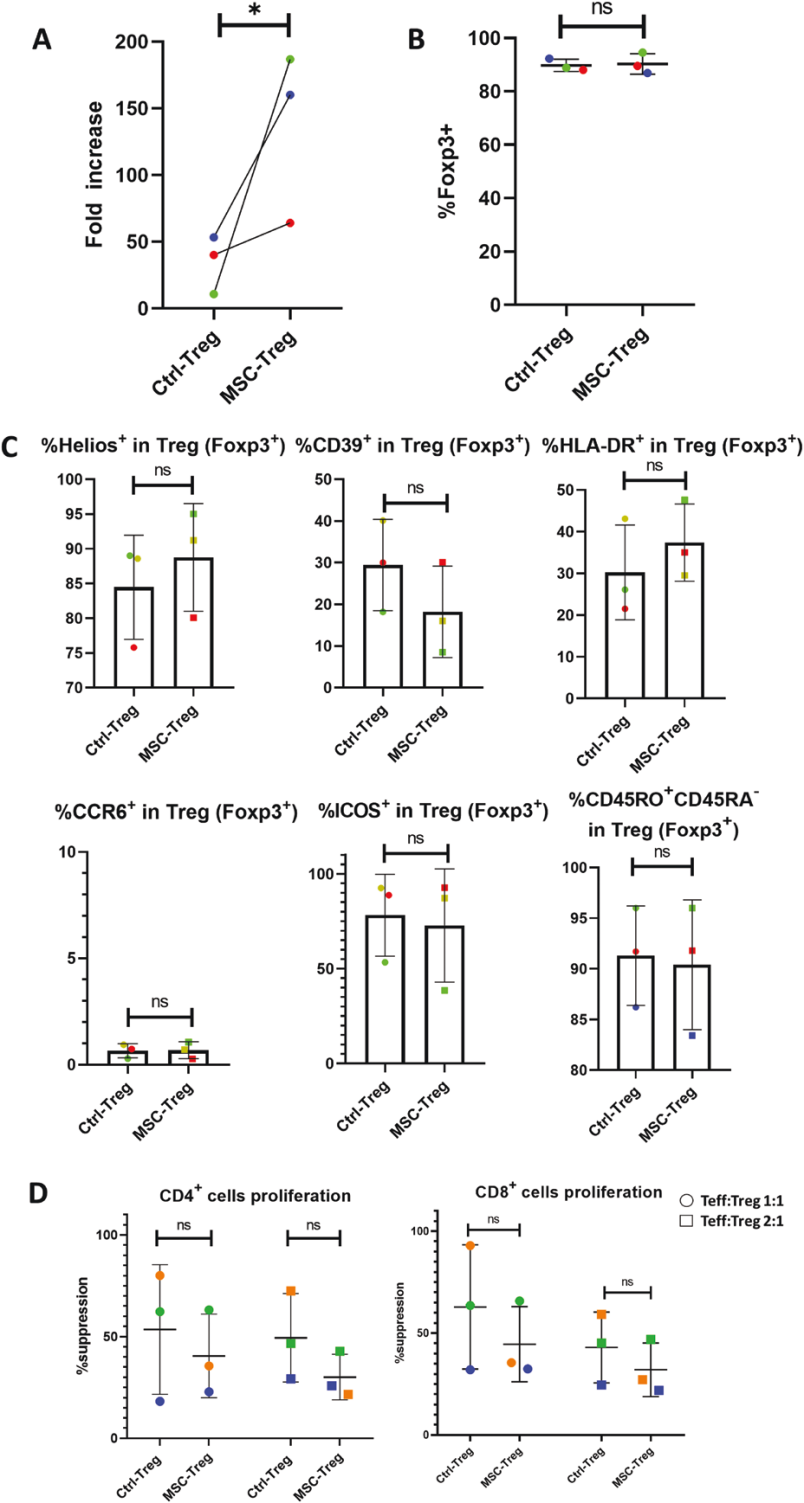
Use of Dynabeads for ex vivo polyclonal regulatory T cell (Treg) expansion confirms that initial coculture with human umbilical cord-derived mesenchymal stromal cells (hUC-MSCs) increases Treg yield and results in high FoxP3 positivity with preservation of other phenotypic and functional properties. Results are shown for human Tregs from *n* = 3 healthy adult donors following Dynabeads activation culture expansion in the absence (Ctrl-Treg) and presence (MSC-Treg) of hUC-MSCs during the first expansion cycle. (A) Graph depicting fold increase in cell number from beginning to completion of Ctrl-Treg and MSC-Treg expansion cultures. (B) Graph depicting %FoxP3^+^ of Ctrl-Treg and MSC-Treg at completion of expansion cultures. (C) Graphs depicting %^+ve^ among the FoxP3^+^ cells in Ctrl-Treg and MSC-Treg for Helios, CD39, CCR6, HLA-DR, ICOS, and CD45RO (%^+ve^ derived from Zombie Red^−^/CD4^+^/Foxp3^+^ cells). (D) Graphs depicting the results of suppression assays in which Ctrl-Treg and MSC-Treg from 3 individual donors were cocultured with polyclonally activated CellTrace Violet (CTV)-labeled primary human CD4^+^ (left) and CD8^+^ (right) T effector cells (Teffs) at Teff:Treg ratios of 1:1 to 8:1. Results are expressed as %suppression of Teff proliferation based on CTV dilution and compared to proliferation of Teffs activated in the absence of Tregs. For all graphs, the results for Tregs from individual donors are distinguished by color, while horizontal strokes and error bars represent mean ± SEM. Statistical analyses for all paired comparisons between Ctrl-Treg and MSC-Treg (A, B, C, D) were performed by Wilcoxon signed-rank test.

As a high level of interindividual variability was observed in Treg yield and %FoxP3^+^ from cultures in which relatively small initial numbers of purified Tregs were activated with MACSiBeads, we performed an additional series of cultures from 3 individual donors using Dynabeads as the primary stimulus (Figure 3). In these cultures, fold expansion was, again, greater for MSC-Treg than for Ctrl-Treg (Figure 3A, 136.9 ± 37.25% vs. 34.7 ± 12.6%), while %FoxP3^+^ was consistently high for both (Figure 3B, 89.7 ± 2.3% vs. 90.3 ± 3.9%). Furthermore, as shown in Figure 3C and D, the proportionate expression of Helios, CD39, HLA-DR, CCR6, ICOS, and CD45RO as well as the suppressive potency against CD4^+^ and CD8^+^ Teffs did not differ between MSC-Treg and Ctrl-Treg.

From this series of experiments, it was concluded that initial coculture with hUC-MSCs consistently increased the yield while preserving key phenotypic and functional properties of Tregs that were polyclonally expanded over 2-3 weeks from relatively small numbers of purified primary blood-derived Tregs. The findings were consistent for cultures using 2 different, clinically relevant expansion bead products (MACSiBeads and Dynabeads), although the latter provided more consistently robust expansion with high %FoxP3^+^. Although adherent MSCs were not observed by microscopic observation by the end of the MSC-Treg cultures (not shown), we formally confirmed by flow cytometry for the MSC surface markers CD90 and CD73, that the proportion of hUC-MSCs among the final cell yield from MSC-Treg cultures was <0.1% (Supplementary Figure S3). The survival and growth of hUC-MSCs in complete Treg expansion medium was compared with their survival and growth in MSC medium and in a 50:50 mix of the 2 media. Microscopic examination and cell counting showed that hUC-MSCs remained viable and adherent with characteristic spindle-shaped morphology in Treg expansion medium for up to 7 days but, in contrast to MSC medium, did not proliferate. In a 50:50 mix of the 2 media, proliferation occurred but to a lesser degree than in 100% MSC medium (Supplementary Figure S4).

### Proportionate expression of CD39, HLA-DR, and ICOS by culture-expanded FoxP3^+^ Treg correlates with potency of suppression of CD4^+^ and CD8^+^ Teff proliferation

**Figure 4.**
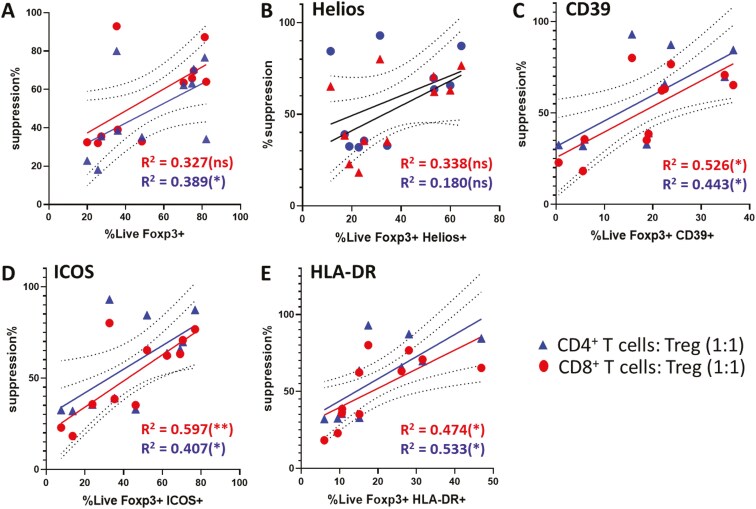
Correlations between proportionate expression of intracellular and surface markers by culture-expanded regulatory T cells (Tregs) and potency of suppression of CD4^+^ and CD8^+^ T effector cells. (A) Scatter plot of the correlations between % expression of FoxP3 among viable cells (%LiveFoxP3^+^) from a total of 11 individual Treg expansion cultures [MSC-Treg (*n* = 4) and Ctrl-Treg (*n* = 3)] and potency of suppression (suppression%) of CD4^+^ (blue triangles) and CD8^+^ (red circles) Teff proliferation at 1:1 Teff:Treg ratio. MSC = mesenchymal stromal cell. (B-E) Similar scatter plots of the correlations between % expression of Helios, CD39, ICOS, and HLA-DR among the Live FoxP3^+^ cells and potency of suppression of CD4^+^ and CD8^+^ Teff proliferation. Analyses were performed by Pearson correlation. ns = not significant; *P < .05, **P < .01.

Given the similar heterogeneity of %FoxP3^+^ cells among the final yields from MSC-Treg and Ctrl-Treg expansion cultures as well as the heterogeneity of %positivity for multiple functionally relevant markers, correlation analyses were performed between these variables and the %suppression of CD4^+^ and CD8^+^ Teffs observed for *n* = 11 of the Treg preparations described in the preceding section. Moderate positive correlations were observed between %Foxp3^+^ and suppressive potency (Figure 4A), which were statistically significant for suppression of CD4^+^ Teffs (*R*^2^ = .389, *P* = .04) but not for CD8^+^ Teffs (*R*^2^ = .327, *P* = .06). For %Helios^+^ among the FoxP3^+^ cells, correlations with suppressive potency for CD4^+^ and CD8^+^ Teffs were not significant (Figure 4B). However, for %CD39^+^, %ICOS^+^, and %HLA-DR^+^ among FoxP3^+^ cells, significant positive correlations were observed for %suppression of both CD4^+^ and CD8^+^ T cells which were stronger than those observed for %FoxP3^+^ alone (Figure 4C-E). Based on these results, we hypothesized that Treg subpopulations with more potent immune suppressive potency are identifiable among polyclonally activated Treg cultures based on specific surface markers and that, for individual donors, these subpopulations may be more effectively expanded by coculture with hUC-MSCs.

### Treg subpopulations defined by expression of CD45RA and HLA-DR have different rates of ex vivo expansion which are enhanced to variable extent by coculture with hUC-MSCs

**Figure 5.**
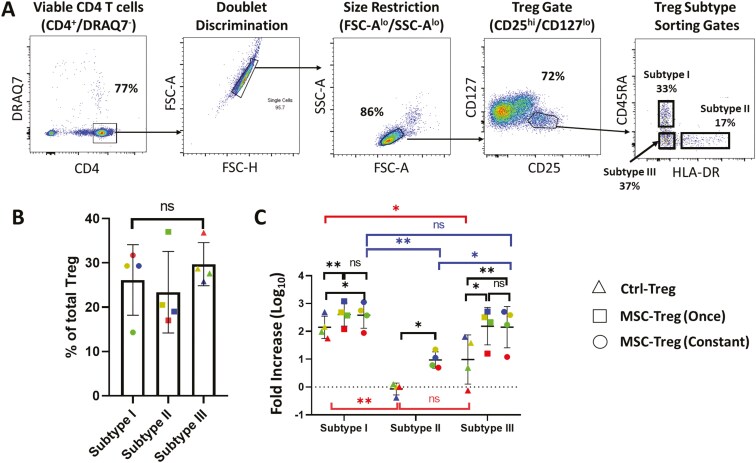
Fluorescence-activated cell sorting (FACS) of 3 human regulatory T cell (Treg) subpopulations with enhanced yields from expansion cultures following human umbilical cord-derived mesenchymal stromal cell (hUC-MSC) coculture. (A) Representative dot plots illustrating a step-by-step gating strategy for FACS purification of 3 Treg subtypes based on surface expression of CD45RA and HLA-DR. (B) Graph depicting the proportions of each of the Treg subtypes among the total FACS-purified Tregs from *n* = 4 healthy donor blood samples. (C) Graph depicting fold increase in cell number of the 3 FACS-purified Treg subtypes at completion of *n* = 4 individual expansion cultures in the absence of hUC-MSCs (Ctrl-Treg), in the presence of hUC-MSCs for the first round of expansion only [MSC-Treg (Once)], or in the presence of hUC-MSCs throughout the culture [MSC-Treg (Constant)]. For all graphs, the results for Tregs from individual donors are distinguished by color, while horizontal strokes and error bars represent mean ± SEM. Statistical analyses were performed by Friedman test (B) and Wilcoxon matched-pairs signed-rank test (C). ns = not significant; *P < .05; **P < .01. Red lines represent comparisons of individual Treg subtypes cultured under Ctrl-Treg conditions. Blue lines represent comparisons of individual Treg subtypes cultured under MSC-Treg (Constant) conditions. Black lines represent comparisons between different culture conditions for each Treg subtypes.

As functional surface marker profiles are known to vary between naive and non-naive Tregs,^44^ a Treg subtype sorting strategy (shown in Figure 5A) was derived, whereby naive (CD45RA^+^/HLA-DR^−^) Tregs were purified as a single population (termed Subtype I), and non-naive (CD45RA^−^) Tregs were purified as 2 separate populations: CD45RA^−^/HLA-DR^+^ (Subtype II) and CD45RA^−^/HLA-DR^−^ (Subtype III) Tregs. The rationale for this sorting strategy was to compare the influences of hUC-MSC coculture on ex vivo expansion of non-antigen-experienced (naive) Tregs with Tregs that had previously encountered an antigenic stimulus (non-naive; effector Tregs) and either did or did not express a surface marker (HLA-DR) that has been consistently reported to correlate with high suppressive potency.^1,27,45^ This sorting strategy was applied to PBMC samples from 4 healthy adult donors for which the proportions of each subtype among total CD4^+^/CD25^hi^/CD127^lo^ Treg are shown in Figure 5B—with the exception of one sample, Subtype II was less frequent than Subtypes I and III.

Following purification by FACS, the 3 Treg subtypes were subjected to ex vivo expansion culture (with polyclonal activation by Dynabeads) using the same protocol as for total Tregs in the absence of hUC-MSCs (Ctrl-Treg), with hUC-MSCs during the first expansion round only [MSC-Treg (Once)—Subtypes I and III only due to limited numbers of Subtype II] or with hUC-MSC coculture maintained throughout the entire expansion culture [MSC-Treg (Constant)]. Cell expansion rates (expressed as log_10_ fold increase) for the 3 Treg subtypes under these 3 culture conditions are summarized for the 4 individual donors in Figure 5C. Notably, under Ctrl-Treg conditions, the fold increases in cell number differed greatly for the 3 subtypes—highest for Subtype I (197.1 ± 198.7), lowest for Subtype II (0.7 ± 0.6), and intermediate for Subtype III (26.8 ± 29.6). For each Treg subtype, both initial and continuous coculture with hUC-MSCs resulted in significantly higher fold increase in cell number, with no differences between MSC-Treg (Once) and MSC-Treg (Constant). However, the magnitude of the enhancing effect of hUC-MSC coculture on fold increase of cell number differed among the subtypes, being substantially greater for Subtypes II and III than for Subtype I. Examples of the microscopic appearances of individual wells of the Treg subtype cultures at the time of initiation and at different timepoints during the first cycle of expansion are shown in Supplementary Figure S5.

### Coculture with hUC-MSCs modulates expression of specific intracellular and surface markers of culture-expanded Treg subpopulations

**Figure 6.**
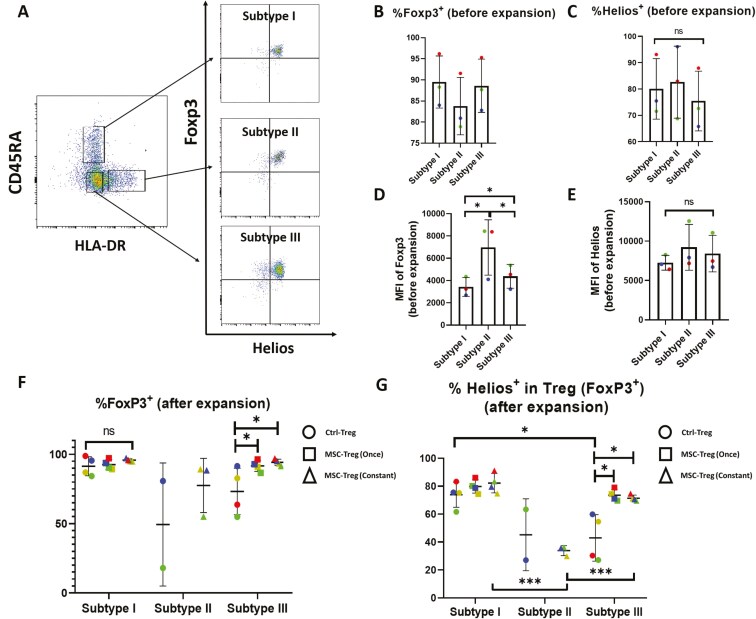
Expression characteristics for Foxp3 and Helios of 3 regulatory T cell (Treg) subpopulations prior to following ex vivo culture expansion in the absence or presence of human umbilical cord-derived mesenchymal stromal cells (hUC-MSCs). (A) Representative flow cytometry dot plots of 3 Treg subpopulations defined on the basis of CD45RA and HLA-DR (left, gated on viable CD4^+^/CD25^+^/CD127^lo^ cells) and subsequently analyzed for expression of FoxP3 and Helios (right, gated on CD45RA^+^/HLA-DR^−^ [Subtype I], CD45RA^−^/HLA-DR^+^ [Subtype II], or CD45RA^−^/HLA-DR^−^ [Subtype III]). (B, C) Graphs depicting % of cells with positive staining for FoxP3 (B) and Helios (C) for each of the Treg subpopulations prior to FACS and ex vivo culture expansion for *n* = 3 individual healthy donor blood samples. (D, E) Graphs depicting the mean fluorescence intensity of the positive staining for FoxP3 (D) and Helios (E) for each of the Treg subpopulations prior to FACS and ex vivo culture expansion for *n* = 3 individual healthy donor blood samples. (F, G) Graphs depicting the percent of cells with positive staining by multicolor flow cytometry for FoxP3 (F) and Helios among FoxP3^+^ cells (G) for 3 Treg subpopulations analyzed at the end of *n* = 4 individual ex vivo expansion cultures in the absence of hUC-MSCs (Ctrl-Treg), in the presence of hUC-MSCs for the first round of expansion only [MSC-Treg (Once)], or in the presence of hUC-MSCs throughout the culture [MSC-Treg (Constant)]. For Subtype II, data were available for Ctrl-Treg and MSC-Treg (Constant) only and for *n* = 2 or 3 donors only as a result of limitations of initial cell number and ex vivo expansion compared to Subtypes I and III. For all graphs, the results for Tregs from individual donors are distinguished by color, while horizontal strokes and error bars represent mean ± SEM. Statistical analyses were performed by Wilcoxon matched-pairs signed-rank test (B, C, D, E) or by paired or unpaired Student’s *t*-test as appropriate (F, G). ns = not significant; *P < .05; **P < .01; ***P < .001.

For the 3 Treg subpopulations, the expression of relevant intracellular and surface proteins was analyzed prior to and/or following ex vivo expansion under different conditions. As shown in Figure 6A-E, the pre-expansion expression characteristics for FoxP3 and Helios [%^+ve^ and mean fluorescence intensity (MFI)] were similar with the exception that the MFI for FoxP3 differed significantly among the subtypes [Figure 6D, Subtype II (6969.0 ± 2480.5) > Subtype III (4376.0 ± 1074.9) > Subtype I (3428.7 ± 848.5)]. In contrast, following culture expansion in the absence of MSCs, %FoxP3^+^ and %Helios^+^ among FoxP3^+^ cells were highest for Subtype I and notably lower for Subtypes II and III (Figure 6F, Ctrl-Treg; representative dot plots are shown in Supplementary Figure S6). However, for Subtype III, initial or continuous coculture with hUC-MSCs resulted in significant increases in FoxP3 and Helios positivity [Figure 6F and G, MSC-Treg (Once) and MSC-Treg (Constant); representative dot plots are shown in Supplementary Figure S6].

**Figure 7.**
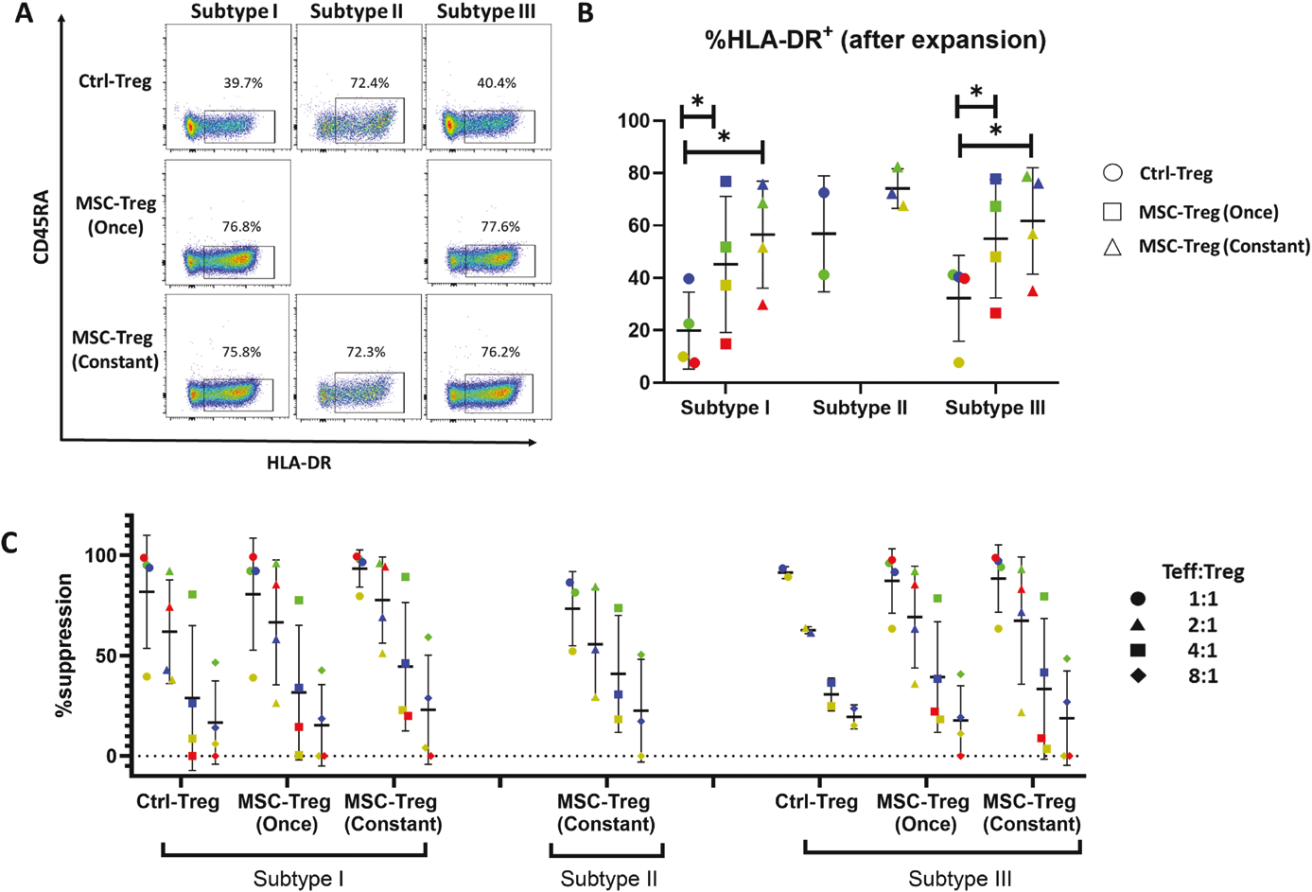
Expression characteristics for HLA-DR (A, B), and potency to suppress CD4^+^ T effector cell proliferation (C) of 3 regulatory T cell (Treg) subpopulations following ex vivo culture expansion in the absence or presence of human umbilical cord-derived mesenchymal stromal cell (hUC-MSCs). (A) Representative flow cytometry dot plots illustrating CD45RA and HLA-DR staining characteristics of 3 Treg subtypes at completion of expansion cultures in the absence of hUC-MSCs (Ctrl-Treg), in the presence of hUC-MSCs for the first round of expansion only [MSC-Treg (Once)], or in the presence of hUC-MSCs throughout the culture [MSC-Treg (Constant)]. The plots shown were gated on viable CD4^+^/CD25^+^/FoxP3^+^ cells. Boxes and percentages indicate CD45RA^−^/HLA-DR^+^ cells. (B) Graph depicting the percent of cells with positive staining by multicolor flow cytometry for HLA-DR for 3 Treg subpopulations analyzed at the end of *n* = 4 individual ex vivo expansion cultures in the absence of hUC-MSCs (Ctrl-Treg), in the presence of hUC-MSCs for the first round of expansion only [MSC-Treg (Once)], or in the presence of hUC-MSCs throughout the culture [MSC-Treg (Constant)]. For Subtype II, data were available for Ctrl-Treg and MSC-Treg (Constant) only and for *n* = 2 or 3 donors only as a result of limitations of initial cell number and ex vivo expansion compared to Subtypes I and III. For all graphs, the results for Tregs from individual donors are distinguished by color, while horizontal strokes and error bars represent mean ± SEM. Statistical analyses were performed by paired Student’s *t*-test or Wilcoxon matched-pairs signed-rank test as appropriate. *P < .05. (C) Graph depicting the results of suppression assays in which preparations of 3 Treg subtypes that were culture-expanded in the absence of hUC-MSCs (Ctrl-Treg), in the presence of hUC-MSCs for the first round of expansion only [MSC-Treg (Once)], or in the presence of hUC-MSCs throughout the culture [MSC-Treg (Constant)] were cocultured with polyclonally activated CellTrace Violet (CTV)-labeled primary human CD4^+^ T effector cells (Teffs) at Teff:Treg ratios of 1:1 to 8:1. In the case of Subtype II, only MSC-Treg (Constant) was successfully expanded for use in suppression assays. Results are expressed as %suppression of Teff proliferation based on CTV dilution and compared to proliferation of Teff activated in the absence of Tregs. For all graphs, the results for Tregs from individual donors are distinguished by color, while horizontal strokes and error bars represent mean ± SEM. Assays were performed for Tregs from *n* = 4 individual donors for all conditions with the exception of Subtype II MSC-Treg (Constant) for which adequate cells were available for *n* = 3 donor samples and of Subtype I Ctrl-Treg for which adequate cells were available for *n* = 2 donor samples. Statistical analyses, performed by Friedman test or Kruskal-Wallis test, did not indicate significant differences among Treg subtypes and culture conditions.

In the case of Class II MHC expression, Subtypes I and III were, by definition, HLA-DR^−^ and Subtype II was HLA-DR^+^. Nonetheless, following culture expansion in the absence of hUC-MSCs (Figure 7A and B, Ctrl-Treg), Subtypes I and III demonstrated moderate proportions of HLA-DR^+^ cells (19.9 ± 14.7% and 32.3% ±16.4%, respectively), while Subtype II remained predominantly but not exclusively HLA-DR^+^ (56.85% ± 22.1%). Of interest, for all 3 Treg Subtypes, initial or continuous coculture with hUC-MSCs resulted in increased proportions of HLA-DR^+^ cells [Figure 7A and B, MSC-Treg (Once) and MSC-Treg (Constant)].

For surface CD39, pre-expansion analysis indicated highest %^+ve^ among Subtype II, but post-expansion %CD39^+^ did not differ among the subtypes or culture conditions. For this marker, there was substantial interindividual variability that tended to remain consistent between pre- and post-expansion Treg subtypes (Supplementary Figure S7A and B). Post-expansion expression characteristics for ICOS and CTLA-4 also demonstrated interindividual variability but did not differ among the Treg subpopulations or expansion culture conditions (Supplementary Figure S8A and B).

From these analyses, it was concluded that the post-expansion expression profiles for key intracellular and surface proteins of the 3 purified Treg subpopulations reflect combined influences of their baseline characteristics, interindividual variabilities, and changes that occur during ex vivo activation and proliferation. In addition, however, coculture with hUC-MSCs either initially or continuously, was found to enhance FoxP3 and Helios positivity among expanded CD45RA^−^/HLA-DR^−^ Tregs in Subtype III and increase MHC class II expression of all Treg subpopulations.

### Tregs subpopulations have comparable suppressive potency against Teff proliferation that is preserved following culture expansion in the presence of hUC-MSCs

For all Treg subpopulation preparations that were successfully expanded under CTRL-Treg, MSC-Treg (Once), and MSC-Treg (Constant) conditions, the suppressive potency against proliferation of polyclonally stimulated Teffs was quantified by flow cytometric analysis of fluorescence dye dilution. As shown in Figure 7C, dose-dependent suppression of CD4^+^ Teff proliferation was observed for culture-expanded Treg Subtypes I, II, and III from *n* = 2-4 individual donors with no clear differences among the subtypes for mean % suppression at Teff:Treg ratios between 1:1 and 1:8. Furthermore, for Subtypes I and III, comparisons of the suppressive potency between CTRL-Treg, MSC-Treg (Once), and MSC-Treg (Constant) revealed no differences. For Subtype II, suppression of Teff proliferation could only be quantified for MSC-Treg (Constant) as purified Subtype II Treg could not be effectively expanded in the absence of hUC-MSC coculture. Similar results were obtained for CD8^+^ Teff suppression assays with culture-expanded Treg subtypes from *n* = 2-3 individual donors (Supplementary Figure S9).

These results confirmed for all 3 Treg subtypes that the higher cell yield associated with hUC-MSC coculture (which, in the case of Subtype II, was necessary for any expansion to occur) is accompanied by preserved Teff suppressive potency. Importantly, this benefit of hUC-MSC coculture is not dependent on the presence of hUC-MSCs throughout the expansion culture. Finally, the results also demonstrated that, for the classical Treg function of suppressing Teff activation, no clear differences in potency were present among the 3 subtypes under any of the culture conditions despite the phenotypic variations described in the preceding section.

## Discussion

The last decade has witnessed important successes in clinical trials that have demonstrated the safety and potential efficacy of intravenously administered, ex vivo expanded Tregs for immune-mediated diseases.^9-11,15,46^ Nonetheless, there remains a need for innovations to improve the consistency and efficiency of Treg manufacture and to support the manufacture of tailored Treg products. For example, in the ONE study, which consisted of a series of trials of immune regulatory cells for kidney transplantation of which the majority were Tregs, 14 of 60 enrolled patients were withdrawn due to cell manufacturing failure.^15^ Similarly, in a trial of Tregs for graft-vs.-host disease prevention, 5 of 13 enrolled patients were withdrawn because the cell number manufactured was lower than required.^17^ The results we present here indicate that coculture with GMP-compatible hUC-MSCs consistently increases the yield of unfractionated Treg from an ex vivo expansion protocol and specifically facilitates the expansion of less numerous FACS-purified Treg subpopulations. In the case of non-naive (CD45RA^−^) Treg subpopulations, meaningful culture expansion was only achieved in the presence of hUC-MSCs. For both naive (CD45RA^+^) and HLA-DR^−^ non-naive Treg subpopulations, the impact of hUC-MSC coculture was similar whether MSCs were present throughout the culture period or only during the initial activation cycle—a finding that is in keeping with Reading et al.’s report of Treg transcriptional reprogramming following MAPC coculture.^33^ Importantly, the phenotypic characteristics and Teff suppressive potency of Tregs expanded in the presence of hUC-MSCs were comparable to those of CTRL-Treg. Our results also demonstrate novel effects of hUC-MSC coculture to promote HLA-DR expression among ex vivo expanded Treg and, potentially, to increase FoxP3 and Helios positivity among expanded non-naive Tregs. Finally, from an efficiency perspective, we demonstrate that Treg/hUC-MSC cocultures activated with Dynabeads allow for more rapid expansion than activation with MACSiBeads, while producing Tregs with similar phenotypes and suppressive potency. Of note, the rates of expansion that were observed in the absence of hUC-MSC coculture for these 2 GMP-compatible T-cell activation products were consistent with those of other studies of human peripheral blood-derived Treg expansion over 2 weeks using either Dynabeads (>100-fold increase)^33,47^ or MACSiBeads (10- to 100-fold increase).^48,49^

Our findings that hUC-MSC coculture specifically increases HLA-DR expression by all 3 Treg subpopulations following expansion is of potential interest to the manufacture of Treg therapies. The mechanisms underlying this observation remain to be determined, but it may reflect the allogeneic nature of the Treg/hUC-MSC cocultures. In keeping with this, Ma et al. reported that bead-expanded human CD4^+^/CD25^+^/CD127^low^ Tregs contained higher proportions of HLA-DR^+^/CD27^+^ cells when concomitantly exposed to irradiated porcine PBMCs compared with either freshly isolated Tregs or Tregs expanded with anti-CD3/CD28 beads alone.^30^ Importantly, primary HLA-DR^+^ Tregs have been considered to be a more stable and suppressive lineage within the total circulating Treg pool, and previous studies have focused on purification and functional analysis of this subpopulation. For example, Baecher-Allan et al. sorted HLA-DR^+^ and HLA-DR^−^ cells from total human blood-derived CD4^+^/CD25^hi^/CD62L^hi^ Tregs and observed that, directly after sorting, HLA-DR^+^ Tregs have higher Foxp3 mRNA and protein expression.^27^ However, following 5 days of in vitro stimulation with anti-CD3/CD2 and IL-2, the FoxP3 mRNA expression of HLA-DR^+^ Tregs was significantly decreased while that of HLA-DR^−^ Tregs was increased. Schaier et al. compared the suppressive potency of 4 Treg subpopulations sorted on the basis of HLA-DR and CD45RA expression from magnetic bead-enriched CD4^+^/CD25^+^/CD127^lo^ blood cells of kidney transplant recipients. In keeping with our own subpopulation analysis, they observed few CD45RA^+^/HLA-DR^+^ Tregs. Following purification of separate HLA-DR^hi^, HLA-DR^lo^, HLA-DR^−^/CD45RA^−^, and HLA-DR^−^/CD45RA^+^ cells, they demonstrated that HLA-DR^hi^ and HLA-DR^lo^ Treg had higher T-cell suppressive potency than either HLA-DR^−^ subpopulation.^45^ Consistent with a more potent and mature phenotype, we report here that purified CD45RA^−^/HLA-DR^+^ Tregs have higher intracellular FoxP3 than both CD45RA^−^/HLA-DR^−^ and CD45RA^+^/HLA-DR^−^ subpopulations but are strikingly resistant to ex vivo expansion—a characteristic that was overcome by coculture with hUC-MSCs. Furthermore, following culture expansion of unfractionated Tregs, HLA-DR expression correlated with % suppression of CD4 and CD8 Teffs. Nonetheless, we also observed that that the CD45RA^−^/HLA-DR^+^ subpopulation had lower proportions of FoxP3 positivity after hUC-MSC-enabled culture expansion and did not exhibit more potent Teff suppressive capacity. Thus, while stromal cell coculture may facilitate the selective expansion of HLA-DR^+^ Tregs that otherwise proliferate poorly in vitro, the potential for this strategy to replicate their higher suppressive potency requires further study. A possible explanation for this experimental outcome is that small numbers of nonsuppressive FoxP3^−^ T cells contained within the initial sorted populations of CD45RA^−^/HLA-DR^+^ Tregs become preferentially expanded in hUC-MSC cocultures. Alternatively, the mechanisms by which hUC-MSCs enable CD45RA^−^/HLA-DR^+^ Tregs to proliferate in response to a polyclonal stimulus may also result in downregulation of FoxP3 and other gene products required for the potent suppressive functions of this subpopulation.

The transcription factor Helios is considered to be a marker of Treg stability.^50^ It is reported to be coexpressed with Foxp3^+^ in the majority (80%-90%) of human Tregs and not to be induced in Tregs differentiated from conventional T cells.^24^ In the current study, we observed that, on average, approximately 80% of Foxp3^+^ Tregs from healthy donors express Helios, and that all 3 Treg subpopulations exhibit similar proportions of FoxP3^+^/Helios^+^ cells at the time of initial isolation. Following culture expansion of unfractionated Tregs, however, we observed no correlation between Helios positivity and Teff suppressive potency. In addition, while the 3 Treg subpopulations we studied demonstrated variable trends in Helios positivity after culture expansion in the absence of hUC-MSCs (high in Subtype I but reduced in Subtypes II and III) as well as with hUC-MSC coculture (restored to high levels in Subtype III), these trends were not associated with clear differences in Teff suppressive potency. Of interest, in a recent study by Lam et al., human naive Tregs with Helios knockdown (by CRISPR/Cas9) were found to have similar phenotype (CTLA-4, Foxp3, conversion to IFN-γ-secreting cells in a high IL-12 environment) and suppressive potency as unedited naive Treg following a 13-day in vitro expansion culture.^51^ This observation would support the conclusion from our current results that preservation of high proportions of FoxP3^+^/Helios^+^ is not necessarily required for the Teff suppressive potency of culture-expanded Treg subpopulations. Nonetheless, the effect of hUC-MSC coculture to prevent the loss of Helios expression while greatly increasing the yield of Subtype III (CD45RA^−^/HLA-DR^−^) Tregs may prove to be beneficial for in vivo stability and/or potency.

The surface protein CD39, which enzymatically converts ATP and ADP to AMP for subsequent conversion to adenosine by CD73, is expressed by a proportion of circulating Tregs and has also been identified as a marker of Treg immune suppressive potency.^52,53^ In a 2015 study, Rissiek et al. sorted CD39^+^ and CD39^−^ subpopulations from human PBMC-derived CD4^+^/CD25^+^/CD127^−^ cells and reported that the CD39^+^ subpopulation had higher proportions of Foxp3^+^ and HLA-DR^+^ cells.^54^ This is consistent with our observed higher proportionate expression of CD39 and FoxP3 by Treg Subtype II (CD45RA^−^/HLA-DR^+^) prior to culture expansion. Of interest, our results also demonstrate that the proportions of CD39^+^ cells, which varied substantially among the individual donor samples, did not much change following ex vivo expansion for the 3 Treg subtypes. Expression of CD39 was also not influenced by hUC-MSC coculture, suggesting that, in contrast to other Treg surface proteins, CD39 expression may represent a stable feature of specific Treg functional states with potential for distinct therapeutic properties. Thus, future studies of the capacity of hUC-MSC coculture to facilitate the expansion of HLA-DR^+^/CD39^+^ primary Treg will be of high interest.

It would be reasonable to question whether the added complexity of a coculture system will have practical feasibility as well as a distinct value proposition for GMP-grade human Treg manufacture in comparison to conventional Treg expansion within a controlled bioreactor.^16^ For the cell combinations we describe here, the feasibility of producing a safe and definable Treg product is supported by the fact that the MSC type used has a strong track record of GMP manufacture and safe administration to human subjects with significant medical illnesses^36,38^; by the fact that FACS-purified human peripheral blood Tregs are also a well-documented source for safe, clinical-grade cell therapies^16^; and by our experimental observations that hUC-MSCs do not robustly proliferate in Treg expansion medium and are required only for the initial round of Treg stimulation. Furthermore, automated GMP-grade bioreactors, such as the Miltenyi CliniMACS Prodigy, which have been successfully used for human Treg expansion, are readily adaptable for culture of both adherent and nonadherent cells.^55,56^ Nonetheless, while technically feasible, translation of our experimental findings to the use of MSC-cocultured Tregs as a treatment for immune-mediated/inflammatory diseases will require further preclinical documentation of distinct, mechanistically defined therapeutic benefits as well as development of cost-effective GMP manufacturing protocols that consistently result in superior yields and purity of the required Treg subpopulations.

In this regard, key mechanistic aspects of the observations we report here remain to be elucidated, and this will be a future goal for the work. Our own prior studies of direct effects of MSCs on T-helper 17 (Th17) cells as well as results reported by others for mechanisms underlying direct interactions between hUC-MSCs and Treg would suggest that cross-talk involving both contact-dependent signals (eg, ICOS and Notch ligands) and inducible release of soluble factors (eg, TGFβ1, prostaglandin E2, and indoleamine 2,3-dioxygenase) is likely to be responsible for the phenomena of enhanced Treg expansion and modulation of specific gene transcripts/proteins.^7,57,58^ Whether hUC-MSCs and Tregs exchange cell surface membrane content or MSCs enhance Treg capacity to modulate antigen-presenting cells through trogocytosis will also be worth investigating.^26^ Furthermore, the mechanistic underpinnings of the differential impact of hUC-MSCs on naive and non-naive Treg subpopulations are of fundamental interest for future investigation. In particular, our observations that purified CD45RA^−^/HLA-DR^+^ Tregs are significantly less susceptible to induced expansion and that this resistance to conventional TCR/IL-2R-mediated proliferation may be overcome by stromal cell contact highlights the current lack of knowledge of the molecular and cellular biology of highly mature human Tregs. A credible hypothesis is that, analogous to CD8 Teffs,^59^ naive, memory, and terminally differentiated Treg subpopulations exhibit variable reliance on antigen-specific/TCR stimulation and cytokine stimuli for survival and proliferation under activating conditions. Thus, in addition to facilitating a translational pathway, detailed mechanistic studies of hUC-MSC cocultures with defined Treg subpopulations will have potential to contribute to fundamental understanding of Treg heterogeneity and immunological roles.

In conclusion, we have demonstrated that initial coculture with clinical-grade hUC-MSCs substantially enhances the yields and preserves the potency of FACS-purified total human Tregs and of specific human Treg subpopulations in ex vivo expansion cultures with the greatest effect on non-naive (CD45RA^−^) subpopulations. This can facilitate the identification, functional characterization, and production of Treg subpopulations with distinct therapeutic benefits and also provides new insights into the influence of MSCs on specific, functionally relevant Treg makers. Our study adds to a growing body of evidence that an important interface exists between the clinical translational pathways of immune modulatory MSCs and Tregs. The further development of our work and that of others toward achieving clinical benefits from MSC-Treg interactions will need to be complemented by experimental studies to elucidate the molecular mechanisms by which MSCs support the expansion of purified Treg subpopulations. It will also be important to determine the broader immune regulatory properties of Treg subpopulations defined by expression of HLA-DR, CD39, and other surface proteins suitable for GMP-compatible purification.

## Supplementary Material

szaf012_suppl_Supplementary_Figures_S1-10_Tables_S1-S3

## Data Availability

The data underlying this article will be shared on reasonable request to the corresponding author.
