# Supplementary material for: Initial or continuous coculture with umbilical cord-derived mesenchymal stromal cells facilitates in vitro expansion of human regulatory T-cell subpopulations
Source: Stem Cells Transl Med. 2025 Jun 14;14(6):szaf012. doi: 10.1093/stcltm/szaf012 (PMC12166524; doi:10.1093/stcltm/szaf012)
Supplement: szaf012_suppl_Supplementary_Figures_S1-10_Tables_S1-S3 [file szaf012_suppl_supplementary_figures_s1-10_tables_s1-s3.pdf]

SUPPLEMENTARY INFORMATION

Initial or continuous co-culture with umbilical cord-derived MSCs facilitates in vitro expansion of human regulatory T cell subpopulations

Running title: MSC co-culture facilitates regulatory T cell expansion

[1] Qifeng Ou, [1] Sarah Cormican, [1] Rachael Power, [1] Sarah Hontz, [2] Shirley A Hanley, [3,4] Md Nahidul Islam, [1] Georgina Shaw, [5] Laura M Deedigan, [5] Emma Horan, [5] Stephen J Elliman, [1,6] Barbara Fazekas, [1,7] Janusz Krawczyk, [1,8] Neema Negi*, [1] Matthew D Griffin*,†

[1] Regenerative Medicine Institute (REMEDI) at CÚRAM SFI Research Centre for Medical Devices, School of Medicine, University of Galway, H19 TK33, Ireland

[2] Flow Cytometry Core Facility, Biomedical Sciences, University of Galway, H19 TK33, Ireland

[3] School of Biological and Chemical Sciences, College of Science and Engineering, University of Galway, Galway, H19 TK33, Ireland

[4] Department of Applied Science, Technological University of the Shannon, Limerick, V94 EC5T, Ireland

[5] Orbsen Therapeutics Ltd., Dangan, Galway, H91 A3EF, Ireland

[6] Biology and Biopharmaceutical Science, Department of Science South East Technological University, Waterford, X91 CF21, Ireland

[7] Haematology Department, University Hospital Galway, Saolta University Healthcare Group, Galway, H91 YR71, Ireland

[8] Department of Chemical Toxicology, Division of Climate and Environment Health, Norwegian Institute of Public Health (Folkehelseinstituttet), Lovisenberggata 8, 0456 Oslo, Norway

* These two authors contributed equally to this work as senior authors

† To whom correspondence should be sent at: Prof. Matthew Griffin, University of Galway, Biomedical Sciences (REMEDI), Corrib Village, Dangan, Galway, Ireland H91 W2TY. Email: matthew.griffin@universityofgalway.ie

Key Words: Cell therapy, cell manufacturing, culture expansion, clinical translation, immunological diseases, mesenchymal stromal cells, regulatory T cells, subpopulations, yield.

Author Contributions

OU: Conceptualization, Data curation, Formal analysis, Investigation, Methodology, Validation, Visualization, Writing – original draft, Writing – review & editing.

SC, SH, MdNI, GS, LD, EH and BF: Conceptualization, Formal analysis, Investigation, Methodology, Writing – review & editing.

RP, SH: Investigation, Methodology, Writing – review & editing.

SJE and JK: Conceptualization, Resources, Supervision, Writing – review & editing.

NN: Conceptualization, Data curation, Formal analysis, Funding Acquisition, Investigation, Methodology, Project administration, Writing – review & editing.

MDG: Conceptualization, Data curation, Funding Acquisition, Methodology, Project administration, Supervision, Validation, Writing – review & editing.

Table of Contents

Supplementary Figure S1 1

Supplementary Figure S2 2,3

Supplementary Figure S3 4

Supplementary Figure S4 5

Supplementary Figure S5 6

Supplementary Figure S6 7

Supplementary Figure S7 8

Supplementary Figure S8 9

Supplementary Figure S9 10

Supplementary Figure S10 11

Supplementary Table S1 12

Supplementary Table S2 12

Supplementary Table S3 13

Supplementary Figure S1

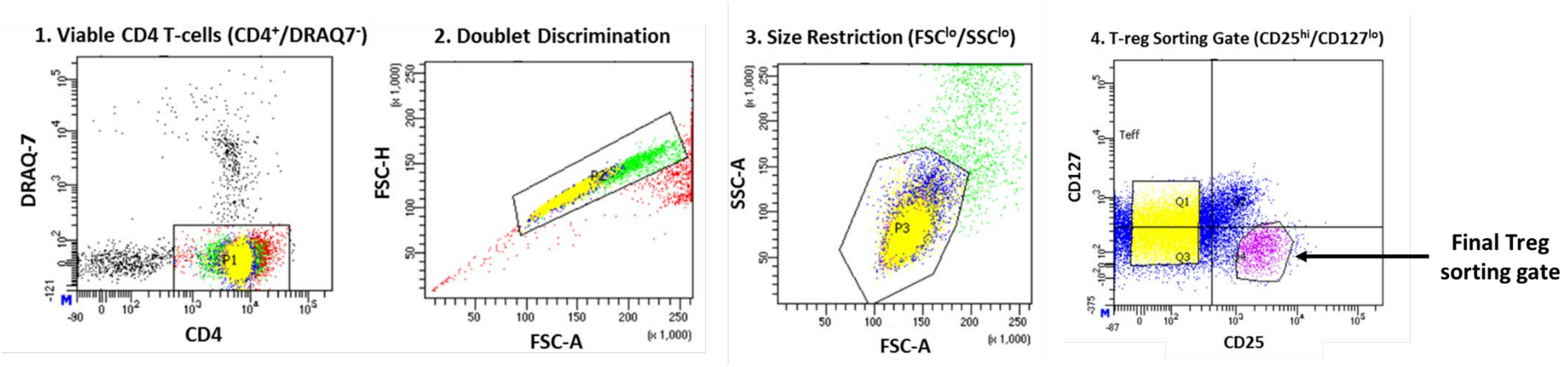

Figure S1: Representative example of the gating strategy for fluorescence-activated cell sorting of Treg from CD4-enriched PBMCs of healthy human volunteers. Sorted Treg were defined as Draq7^{-}CD4^{+}CD25^{high}CD127^{low} cells.

Supplementary Figure S2

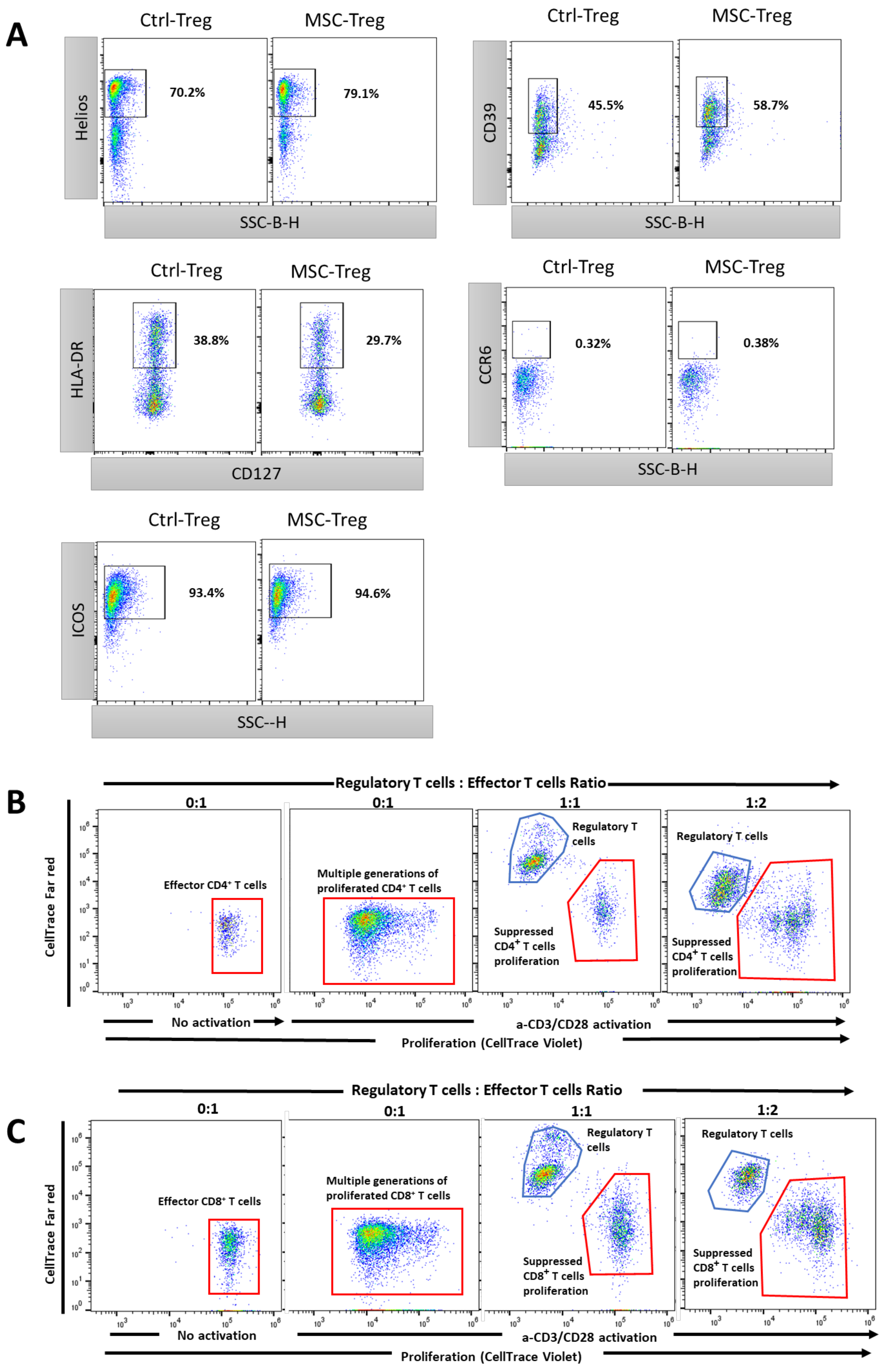

Figure S2: A. Representative flow cytometry dot plots of $\%^{+ve}$ among the $FoxP3^{+}$ cells in Ctrl-Treg and MSC-Treg from 4 individual donors for Helios, CD39, CCR6, HLA-DR and ICOS ($\%^{+ve}$ derived from Zombie red-/$CD4^{+}$/$Foxp3^{+}$ cells). **B & C.** Representative flow cytometry gating strategies depicting the results of suppression assays in which CellTrace Far Red-labelled Ctrl-Treg and MSC-Treg were co-cultured with polyclonally-activated, CelTrace Violet(CTV)-labelled primary human $CD4^{+}$ (**A**) and $CD8^{+}$ (**B**) T effector cells (Teff) at Teff:Treg ratios of 1:1 and 2:1.

Supplementary Figure S3

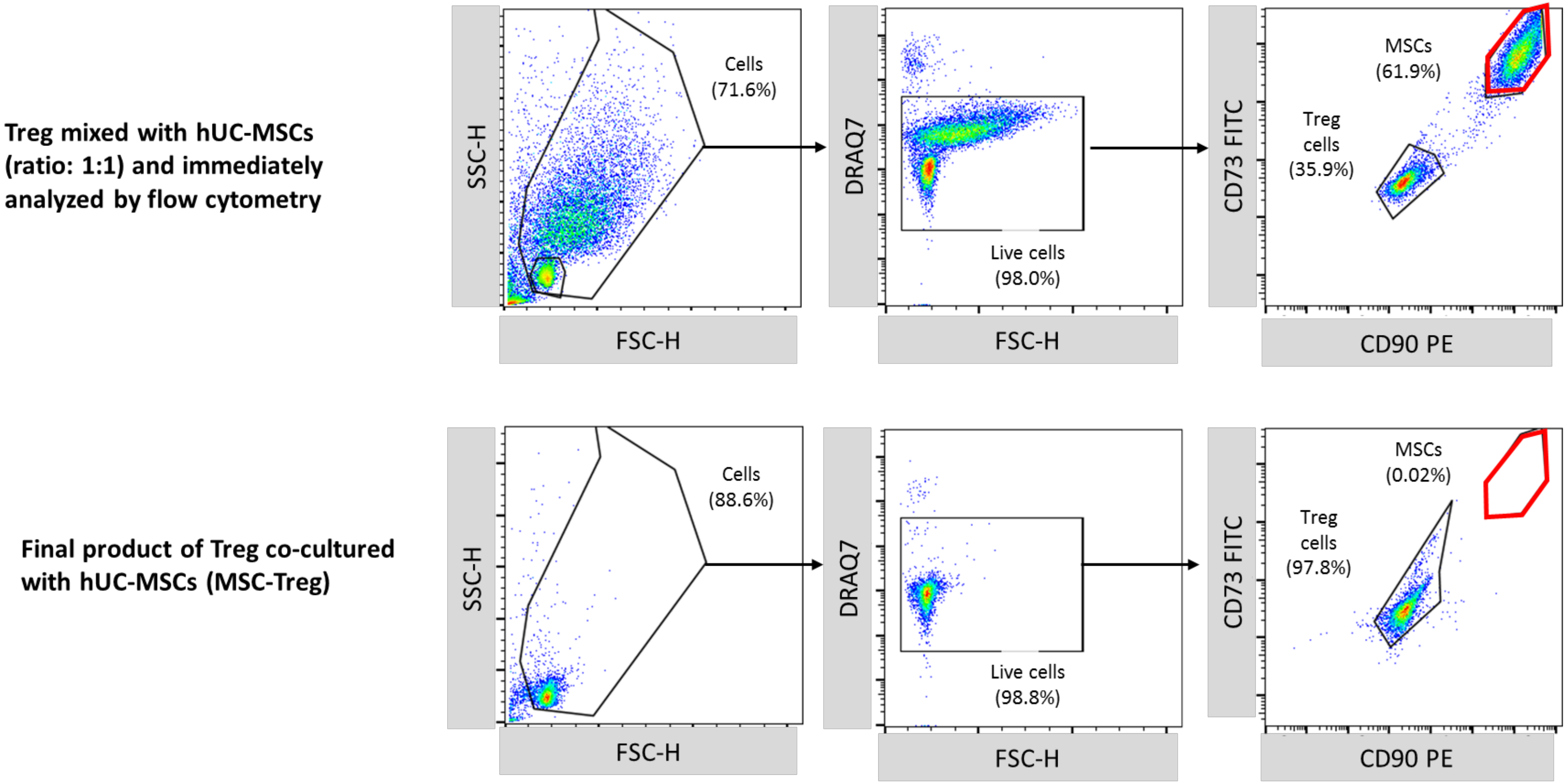

Figure S3: Flow cytometry dot plots demonstrating the proportions of MSCs (CD90$^+$/CD73$^+$) and T cells (CD90$^-$/CD73$^-$) among *Upper*: freshly-prepared admixture of human umbilical cord-derived MSCs (hUC-MSCs) and culture-expanded regulatory T cells (Treg) and Lower: Cells collected at the end of a 2 week expansion culture in which primary Treg were co-cultured with hUC-MSCs (MSC-Treg). The proportion of MSCs detected among the MSC-Treg sample was 0.02%.

Supplementary Figure S4

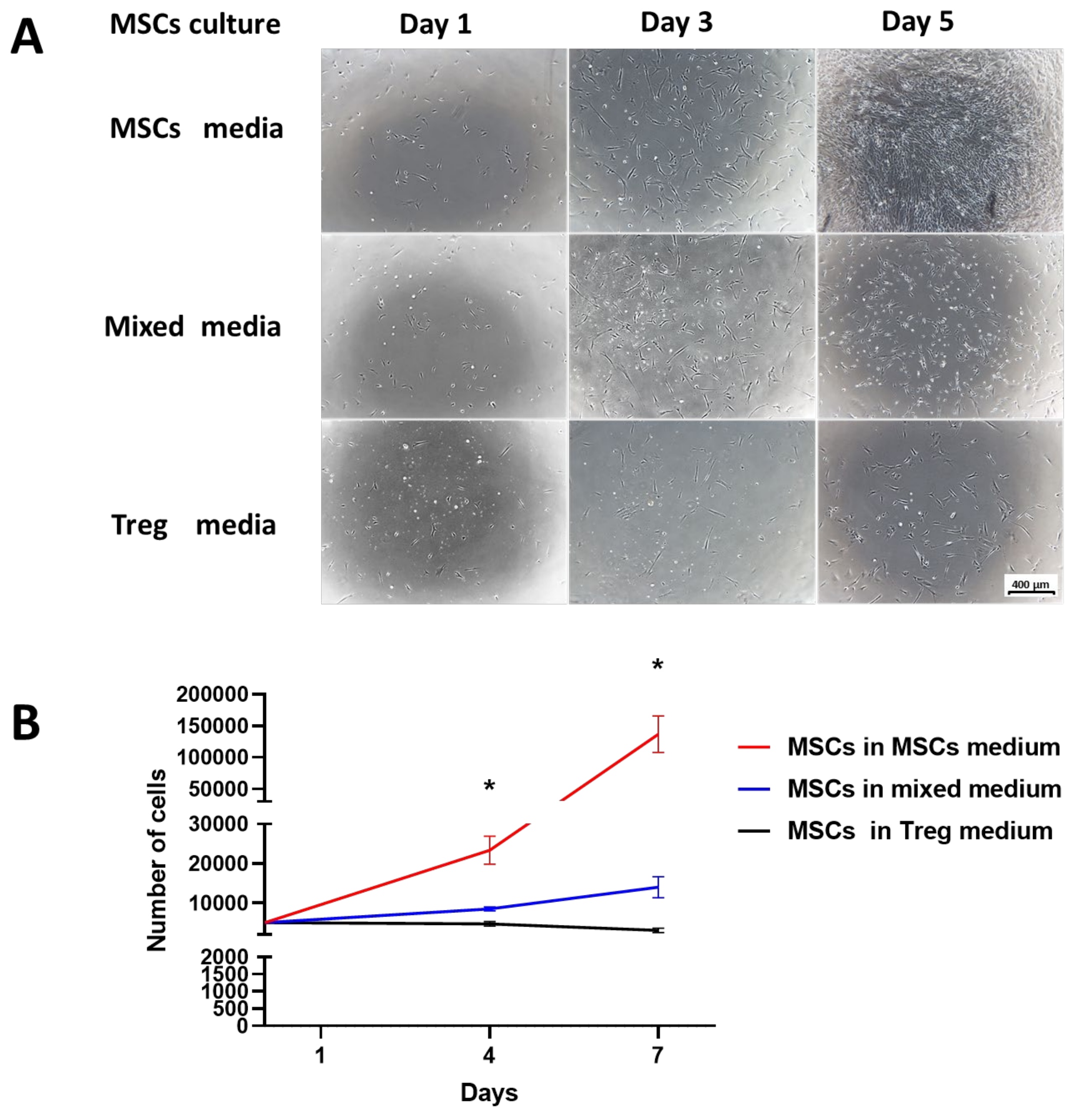

Figure S4 Representative figures (A) and quantitative analysis (B) showing cell number of MSCs in different media. A total of 5000 hUC-MSCs were seeded in each well of a 24-well plate and cultured under one of three conditions: MSCs medium, Treg medium, or a mixed medium (1:1 ratio of MSCs medium and Treg medium). Cell numbers were evaluated on Days 0, 4, and 7, with three technical replicates performed for each condition. The MSC medium was prepared using MEM-α medium supplemented with 10% fetal bovine serum (FBS) and 1% penicillin-streptomycin. The Treg medium was Treg expansion medium formulated by TexMACS™ Medium supplemented with 5% human AB serum, 1% penicillin-streptomycin, 500 IU/mL human recombinant IL-2, and 200 nmol/mL rapamycin. (*: p<0.01)

Supplementary Figure S5

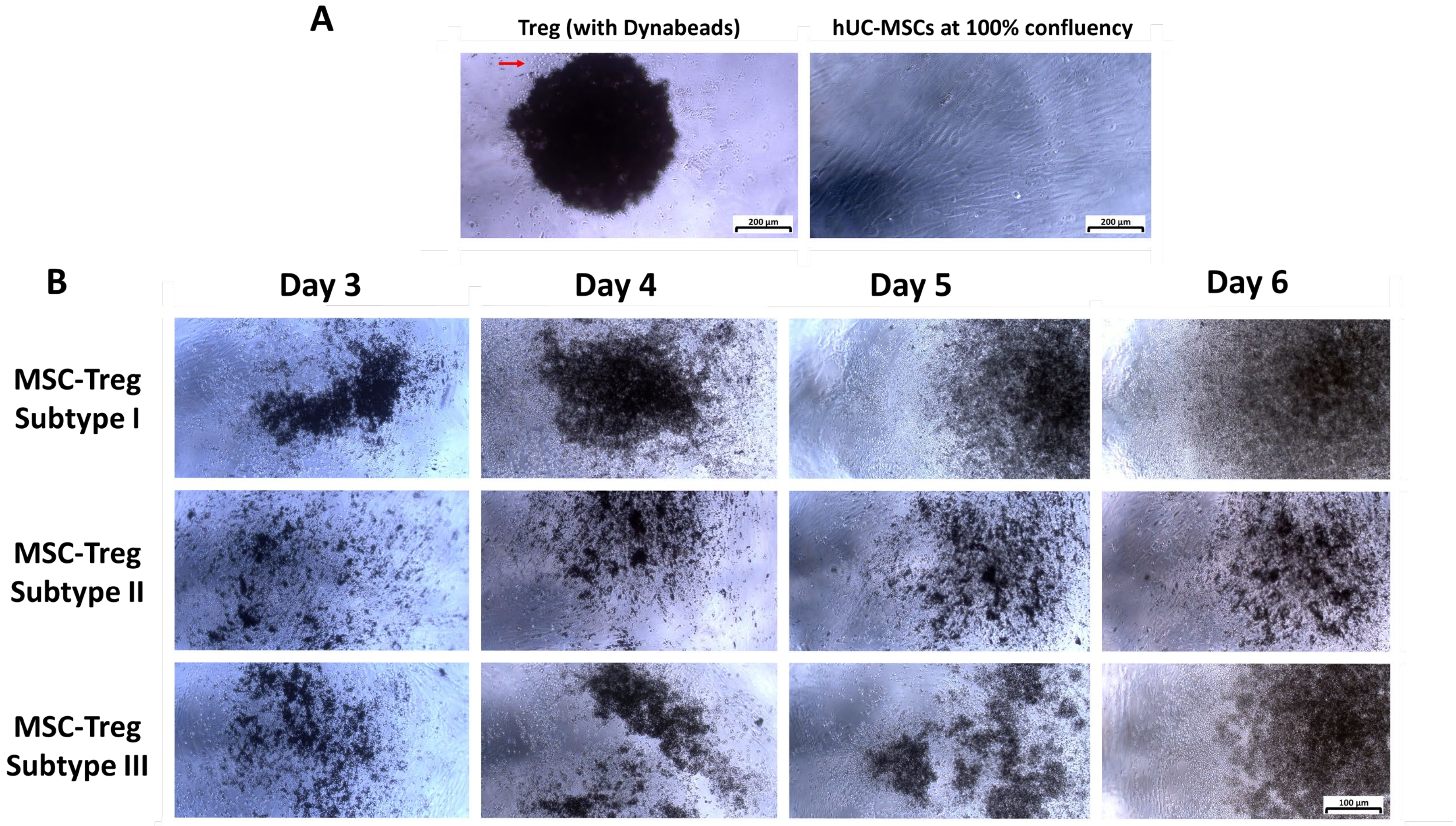

Figure S5: A. Representative photomicrographs of individual culture wells of Treg activated with Dynabeads in the absence of hUC-MSCs (*left*) and of hUC-MSCs cultured alone to confluency (*Right*). Representative photomicrographs of individual wells of purified Treg Subtypes I, II and III co-cultured with hUC-MSCs and activated with Dynabeads for 3, 4, 5, and 6 days. The relative abundances of the three Treg subtypes (Subtype I > Subtype III > Subtype II) is visually evident by day 6.

Supplementary Figure S6

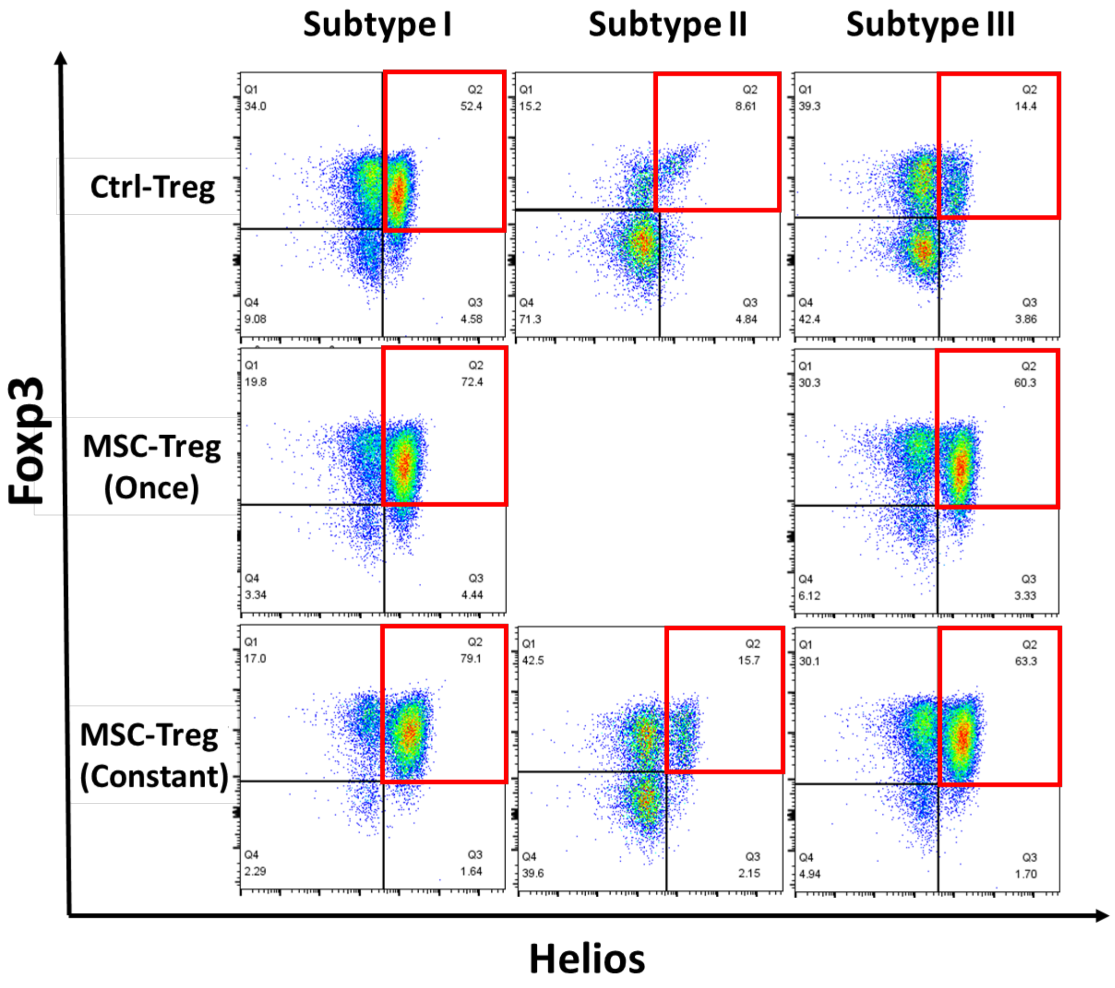

Figure S6: Representative flow cytometry dot plots illustrating FoxP3 and Helios staining characteristics of three Treg subtypes at completion of expansion cultures in the absence of hUC-MSCs (Ctrl-Treg), in the presence of hUC-MSCs for the first round of expansion only [MSC-Treg (Once)] or in the presence of hUC-MSCs throughout the culture [MSC-Treg (Constant)]. The plots shown were gated on viable CD4+/CD25+/FoxP3+ cells.

Supplementary Figure S7

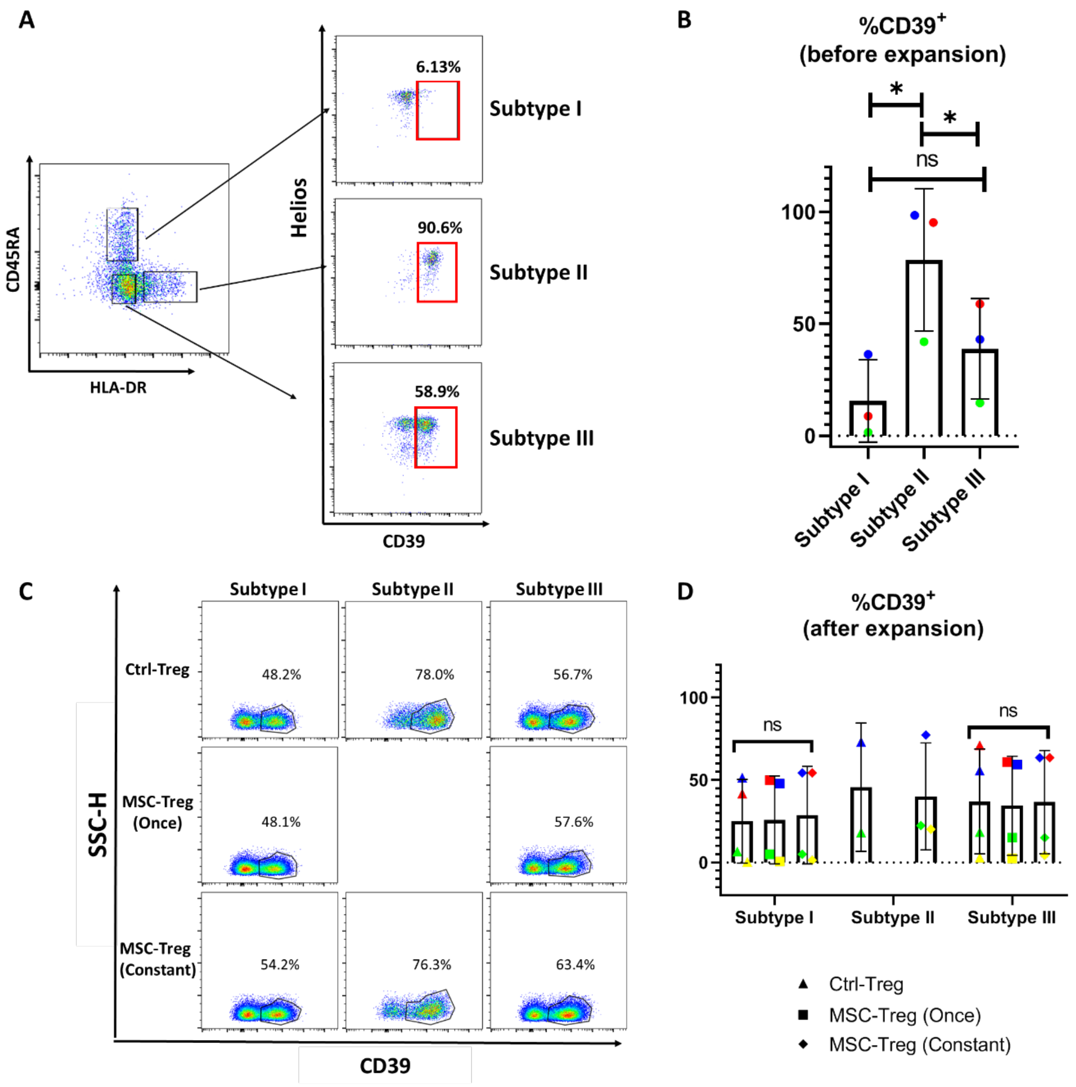

Figure S7: A. Representative flow cytometry dot plots of three Treg subpopulations defined on the basis of CD45RA and HLA-DR (left, gated on viable $CD4^+/CD25^+/CD127^{lo}$ cells) and subsequently analyzed for expression of Helios and CD39 (right, gated on $CD45RA^+/HLA\text{-}DR^-$ (Subtype I), $CD45RA^-/HLA\text{-}DR^+$ (Subtype II) or $CD45RA^-/HLA\text{-}DR^-$ (Subtype III). **B.** Graph depicting $\%CD39^+$ of each of the Treg subpopulations prior to fluorescence-activated cell sorting and ex vivo culture expansion for n=3 individual healthy donor blood samples. **C.** Representative flow cytometry dot plots indicating $\%CD39^+$ of three Treg subtypes at completion of expansion cultures in the absence of hUC-MSCs (Ctrl-Treg), in the presence of Huc-MSCs for the first round of expansion only [MSC-Treg (Once)] or in the presence of hUC-MSCs throughout the culture [MSC-Treg (Constant)]. The plots shown were gated on viable $CD4^+/CD25^+/FoxP3^+$ cells. **D.** Graph depicting $\%CD39^+$ of each of the Treg subpopulations for n=4 healthy donor samples following ex vivo culture expansion in the absence of hUC-MSCs (Ctrl-Treg), in the presence of hUC-MSCs for the first round of expansion only [MSC-Treg (Once)] or in the presence of hUC-MSCs throughout the culture [MSC-Treg (Constant)]. For all graphs, the results for Treg from individual donors are distinguished by color. Statistical analyses were performed by Wilcoxon matched-pairs signed-rank test (B) and Mann-Whitney U test (D) ns: not significant; *: $p<0.05$

Supplementary Figure S8

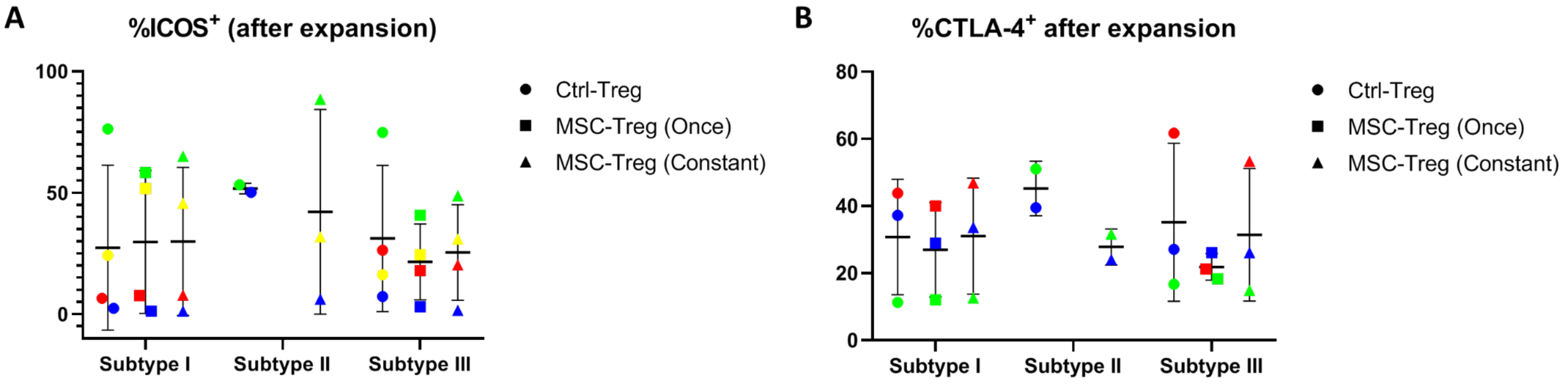

Figure S8: Graphs depicting the percent of cells with positive staining by multi-color flow cytometry for ICOS (**A**) and CTLA-4 (**B**) among three Treg subpopulations analysed at the end of ex vivo expansion cultures in the absence of hUC-MSCs (Ctrl-Treg), in the presence of hUC-MSCs for the first round of expansion only [MSC-Treg (Once)] or in the presence of hUC-MSCs throughout the culture [MSC-Treg (Constant)]. The data for n=4 individual healthy donor samples are expressed as % positive for the marker in question among viable $CD4^+/CD25^+/FoxP3^+$ cells. For all graphs, the results for Treg from individual donors are distinguished by color. For Subtype II, data were available for Ctrl-Treg and MSC-Treg (Constant) only and for n=2 or n=3 donors only as a result of limitations of initial cell number and of ex vivo expansion compared to Subtypes I and III. No significant differences were present by statistical analyses of data based on Treg subtypes or culture condition.

Supplementary Figure S9

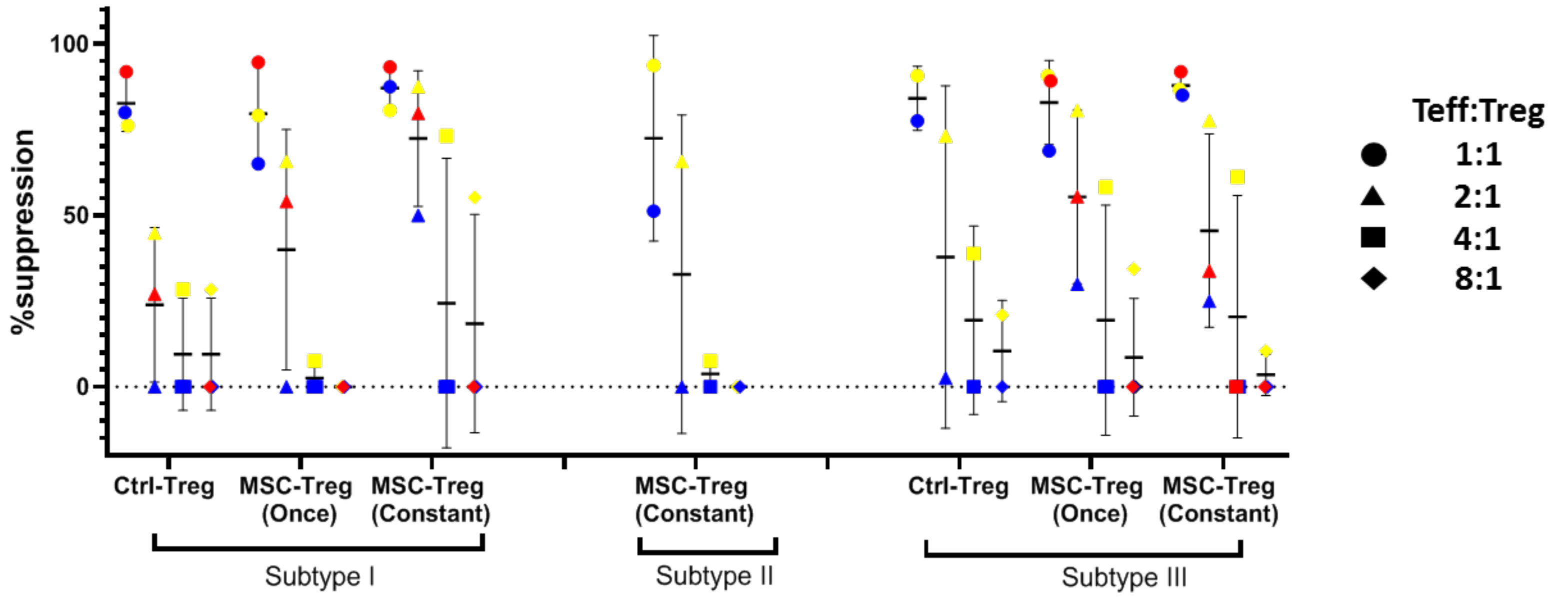

Figure S9: Graph depicting the results of suppression assays in which preparations of three Treg subtypes that were culture-expanded in the absence of hUC-MSCs (Ctrl-Treg), in the presence of hUC-MSCs for the first round of expansion only [MSC-Treg (Once)] or in the presence of hUC-MSCs throughout the culture [MSC-Treg (Constant)] were co-cultured with polyclonally-activated, CellTrace Violet(CTV)-labelled primary human $CD8^{+}$ T effector cells (Teff) at Teff:Treg ratios of 1:1 and 2:1. In the case of Subtype II, only MSC-Treg (Constant) were successfully expanded for use in suppression assays. Results are expressed as %suppression of Teff proliferation based on CTV dilution and compared to proliferation of Teff activated in the absence of Treg. For all graphs, the results for Treg from individual donors are distinguished by color while horizontal strokes and error bars represent mean ± SEM. Assays were performed for Treg from n=4 individual donors for all conditions with the exception of Subtype II MSC-Treg (Constant) for which adequate cells were available for n=3 donor samples and of Subtype I Ctrl-Treg for which adequate cells were available for n=2 donor samples. Statistical analyses, performed by Friedman test or Kruskal–Wallis test did not indicate

Supplementary Figure 10

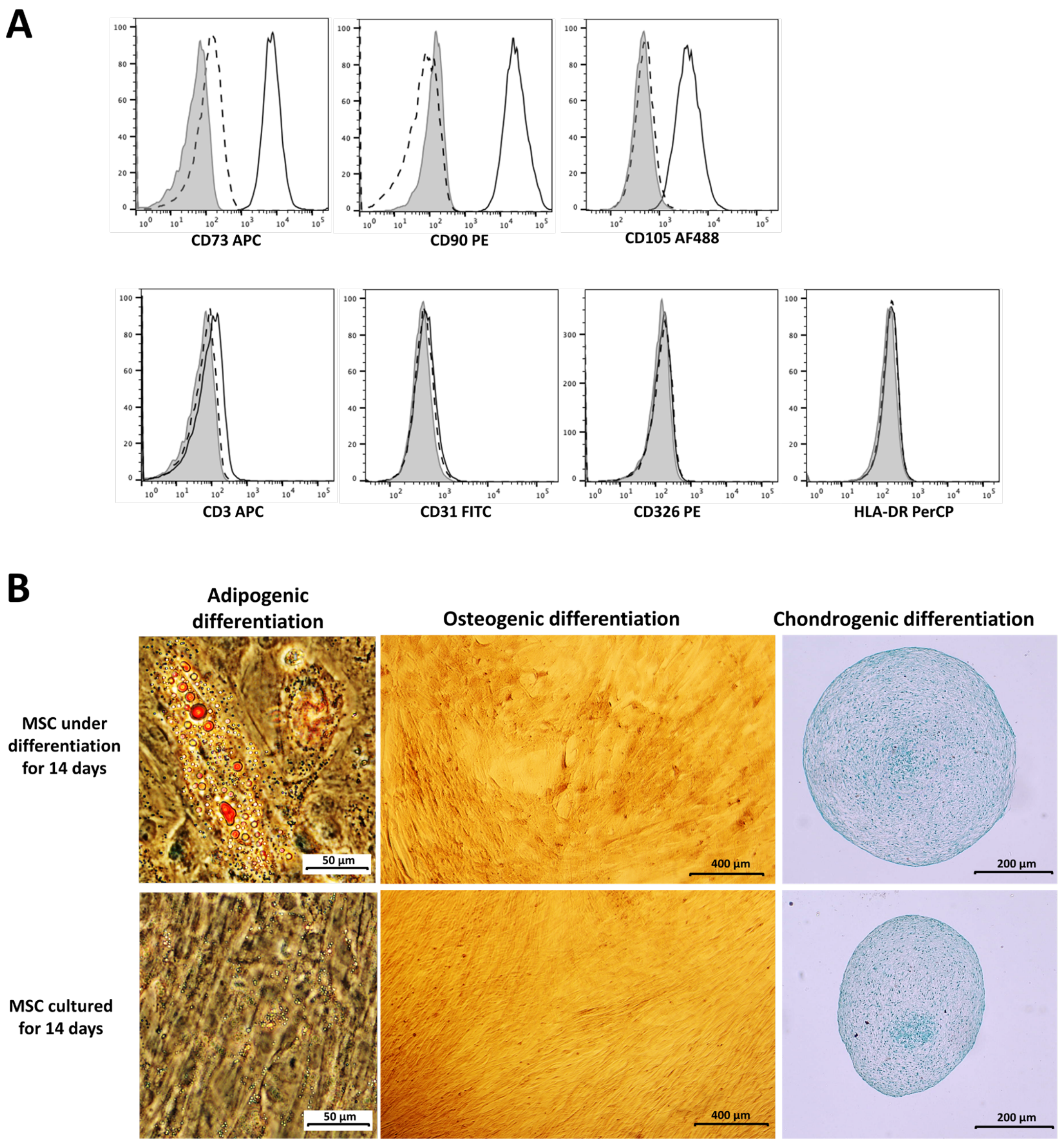

Figure S10. *Characterization of hUC-MSCs used in present study.* **A**. Flow cytometric histogram showing the surface markers on the hUC-MSCs (solid black line) and their isotype control and fluorescence minus one control (dashed black and shaded histogram). **B.** Microscopic observation of trilineage differentiation. Representative figures show Oil red O staining for adipogenic differentiation, Alizarin red staining for osteogenic differentiation, Alcian blue staining for chondrogenic differentiation (from left to right). hUC-MSCs of passage 6 was cultured in different differentiation medium for 2 weeks before the measurement.

Supplementary Table S1: Summary of the numbers and proportions of PBMCs, $CD4^+$ T cells and Treg from seven separate T-reg sorting experiments.

Experiment Number (Donor)	Total PBMC Number	Enriched $CD4^+$ T cell Number	Sorted Treg Number	% Treg among total CD4+ cells	% Treg among total PBMCs
1	70×10^6	15.0×10^6	6.0×10^5	4.00%	0.80%
2	8×10^6	2.8×10^6	1.1×10^5	3.90%	1.30%
3	40×10^6	8.0×10^6	3.0×10^5	4.20%	0.70%
4	33×10^6	10.0×10^6	3.4×10^5	3.40%	1.00%
5	55×10^6	20.0×10^6	6.0×10^5	3.00%	1.10%
6	60×10^6	20.0×10^6	6.0×10^5	3.00%	1.00%
7	48×10^6	14.0×10^6	4.4×10^5	3.10%	0.90%
Mean ± SD	44.9 ± 20.4	12.8 ± 6.3	4.3 ± 1.9	3.5% ± 0.5%	0.97% ± 0.19%

Supplementary Table S2: Details for Treg expansion protocol with or without initial hUC-MSC co-culture**.**

	T-reg cell (counts/well)	hUC-MSCs (cells/well)	IL-2 (IU/ml)	Rapamycin (nM)	Bead:Cell Ratio	Medium exchange interval	Duration of expansion	Number of splits
**Ctrl-Treg**	12,500/25,000	0	1000	100	4:1	2 days	18-21days	1-3
**MSC-Treg**	12,500/25,000	5000	1000	100	4:1	2 days	21 days	2-5

Supplementary Table S3 List of flow cytometric antibodies

Target	Fluorochrome	Vendor	Catalog #	Clone	Target Species
Live/dead viability	Zombie Red Fixable	Biolegend	423110	NA	
	7-AAD	Biolegend	420404		
	Draq7	Invitrogen	D15106		
CD4	FTIC	Biolegend	300506	RPA-T4	Human
CD25	Pe-Cy7	Invitrogen	25-0259-42	BC96	Human
CD127	Brilliant Violet 785	Biolegend	351330	A019D5	Human
CD127	PE	Invitrogen	12-1278-42	eBioRDR5	Human
HLA-DR	eFluor450	Invitrogen	48-9952-42	L243	Human
CD39	PerCP-eFluor 710	Invitrogen	46-0399-42	eBioA1	Human
ICOS	BV510	Biolegend	313525	C398.4A	Human
CCR6	PE	Invitrogen	12-1969-42	R6H1	Human
CD45RA	APC	Invitrogen	17-0458-42	HI100	Human
CD45RO	PerCP	Biolegend	304252	UCHL1	Human
	BV510	Biolegend	304246	UCHL1	Human
Helios	PE-eFluor@610	Invitrogen	61-9883-42	22F6	Human
Foxp3	R718	BD Biosciences	566935	259D/C7	Human
CTLA-4	Brilliant Ultra Violet 805	Invitrogen	368-1529-42	14D3	Human
Celltrace Violet	Violet	Invitrogen	C34571	NA	
Celltrace Far Red	Far Red	Invitrogen	C34572		
CD90	PE	Biolegend	328110	5E10	Human
CD73	FITC	Biolegend	344016	AD2	Human
